# Graduates of different UK medical schools show substantial differences in performance on MRCP(UK) Part 1, Part 2 and PACES examinations

**DOI:** 10.1186/1741-7015-6-5

**Published:** 2008-02-14

**Authors:** IC McManus, Andrew T Elder, Andre de Champlain, Jane E Dacre, Jennifer Mollon, Liliana Chis

**Affiliations:** 1 Department of Psychology, University College London, Gower Street, London WC1E 6BT, UK; 2 Royal College of Physicians of Edinburgh, 9 Queen Street, Edinburgh EH2 1JQ, UK; 3 National Board of Medical Examiners, 3750 Market Street. Philadelphia, PA 19104-3102, USA; 4 Academic Centre for Medical Education, University College London, Holborn Union Building, Archway Campus, Highgate Hill, London N19 5LW, UK; 5 MRCP(UK) Central Office, Examinations Department, 11 St. Andrews Place, Regent's Park, London NW1 4LE, UK

## Abstract

**Background:**

The UK General Medical Council has emphasized the lack of evidence on whether graduates from different UK medical schools perform differently in their clinical careers. Here we assess the performance of UK graduates who have taken MRCP(UK) Part 1 and Part 2, which are multiple-choice assessments, and PACES, an assessment using real and simulated patients of clinical examination skills and communication skills, and we explore the reasons for the differences between medical schools.

**Method:**

We perform a retrospective analysis of the performance of 5827 doctors graduating in UK medical schools taking the Part 1, Part 2 or PACES for the first time between 2003/2 and 2005/3, and 22453 candidates taking Part 1 from 1989/1 to 2005/3.

**Results:**

Graduates of UK medical schools performed differently in the MRCP(UK) examination between 2003/2 and 2005/3. Part 1 and 2 performance of Oxford, Cambridge and Newcastle-upon-Tyne graduates was significantly better than average, and the performance of Liverpool, Dundee, Belfast and Aberdeen graduates was significantly worse than average. In the PACES (clinical) examination, Oxford graduates performed significantly above average, and Dundee, Liverpool and London graduates significantly below average. About 60% of medical school variance was explained by differences in pre-admission qualifications, although the remaining variance was still significant, with graduates from Leicester, Oxford, Birmingham, Newcastle-upon-Tyne and London overperforming at Part 1, and graduates from Southampton, Dundee, Aberdeen, Liverpool and Belfast underperforming relative to pre-admission qualifications. The ranking of schools at Part 1 in 2003/2 to 2005/3 correlated 0.723, 0.654, 0.618 and 0.493 with performance in 1999–2001, 1996–1998, 1993–1995 and 1989–1992, respectively.

**Conclusion:**

Candidates from different UK medical schools perform differently in all three parts of the MRCP(UK) examination, with the ordering consistent across the parts of the exam and with the differences in Part 1 performance being consistent from 1989 to 2005. Although pre-admission qualifications explained some of the medical school variance, the remaining differences do not seem to result from career preference or other selection biases, and are presumed to result from unmeasured differences in ability at entry to the medical school or to differences between medical schools in teaching focus, content and approaches. Exploration of causal mechanisms would be enhanced by results from a national medical qualifying examination.

## Background

The Education Committee of the General Medical Council (GMC), in its wide-ranging report of June 2006, *Strategic Options for Undergraduate Education in the United Kingdom *[1], highlighted the lack of information available to assess whether graduates from different UK universities vary significantly in the knowledge, skills or behaviours which are likely to be relevant to their future competence or performance as doctors. If graduates of different medical schools were to perform differently, then many important questions would be raised about the causes of the variation, with perhaps the most important question concerning the extent to which differences in teaching methods and provision are the causal origin of performance differences. At present the UK does not have a national medical licensing examination, in contrast to the situation in the USA [1], and the GMC has implied that differences in medical school performance would be a strong argument for the benefits of introducing such an examination. The more recent Tooke Report of October 2007 has also argued, more strongly, that "a national test of knowledge" should be introduced at undergraduate level in UK medical schools, saying that "A national examination would ... encourage development within medical schools, serve as a safeguard when medical schools are developing new curricula, and ensure core knowledge and skills are taught and assessed ([2], p. 126)." In the absence of a national licensing examination, the performance of medical graduates in existing postgraduate assessments, particularly those occurring early in the postgraduate career, is one of the few valid sources of information for assessing differences between graduates of different UK medical schools.

The Membership of the Royal Colleges of Physicians (MRCP(UK)) examination is a three-stage, high-stakes, international postgraduate medical assessment, the completion of which forms a critical part of career progression for aspiring physicians in the UK, and is attempted by about 30% of all UK medical graduates. Medical graduates from UK universities and elsewhere sit the first part of the examination as early as 18 months after graduation and most complete the third and final part within a further 3 years. The format of the examination has been described in detail elsewhere [3-8], and details, example questions, marking schemes, etc., can be found at the examination website [9]. Briefly, the examination consists of three parts. Part 1 and Part 2, which are taken sequentially, both consist of best-of-five multiple choice examinations, with Part 1 concentrating on diagnosis, basic management and basic medical science, while Part 2 has longer questions involving more complex data interpretation, including photographic and other visual material, and considers more in-depth issues of diagnosis and management within internal medicine. Both examinations are blue-printed to cover the typical range of acute and chronic conditions presenting in the wide range of patients seen in general medical practice, and the diagnostic, therapeutic and management options which need to considered. The pass mark is set by Angoff-based criterion-referencing coupled with a Hofstee procedure. The third part of the examination, Part 2 Clinical (PACES), is a clinical examination, similar in some ways to an OSCE, in which candidates rotate around five 20-minute stations, seeing a range of patients and simulated patients, typically two or more at each station, and the candidates are required to interview, examine and discuss management options. Two stations are devoted to communication, with one emphasizing the taking of history and the communication of technical information and the other looking at more difficult communication problems such as breaking bad news or asking permission to take organs for transplantation. Each candidate on each case is assessed separately and independently by two trained examiners, with different examiners at each station. PACES can only be taken after Part 1 and Part 2 have both been passed.

In this paper we examine candidate performance in the three components of the MRCP(UK) for variation in performance in relation to the institution of graduation. The *main analysis *is a multilevel model assessing whether graduates from different UK medical schools performed differently in the three components of MRCP(UK) from 2003 to 2005, with differences between medical schools then being assessed in relation to 'compositional variables' describing the schools. An *additional analysis *looks at a much longer time series of data for performance on the Part 1 examination only from 1989 to 2002.

## Methods

### Main analysis

The primary data for the analysis were retrieved from the MRCP(UK) database. Candidates included in the *main analysis *had taken one or more parts of the examination in eight consecutive diets between 2003/2 and 2005/3 (where 2005/3, for instance, indicates the third diet of 2005, with three diets per year of each part of the exam).

### Additional analysis

The data for the *additional analysis *were for Part 1 only from 1989/1 to 2003/1, as well as Part 1 data from 2003/2 to 2005/3.

Candidates were only included who had graduated from one of the 19 UK universities then awarding medical degrees. The formats of the Part 1, Part 2 and PACES stages of the examination were stable between 2003/2 and 2005/3. The Part 1 examination comprised two separate 3-hour papers each of 100 test items in a one-answer-from-five (best-of-five) format. The written examination of Part2 comprised two separate 3-hour papers each of 100 questions in a one-answer-from-five (best-of-five) format until the last 2003 diet when it increased to three 3-hour papers each of 90 questions. The PACES examination comprised a five-station, structured clinical examination lasting 2 hours, incorporating 10 separate clinical encounters each of which was directly observed and assessed by two different and experienced clinician examiners, with each candidate being assessed by 10 examiners in total. There were three diets of Part 1, Part 2 and PACES each year. From 1989/1 to 2002/1 the Part 1 examination consisted of a single paper containing 300 multiple true-false items. From 2002/2 to 2003/1 the Part 1 exam consisted of a similar multiple true-false paper and a separate best-of-five exam with 100 questions.

### Examination scores

Results for all three parts are reported as a percentage score. For Part 1 and Part 2 the pass mark since 2002/2 has been criterion-referenced, whereas for PACES the examination is implicitly criterion-referenced and the pass mark set at 41 out of 56 for all diets [10]. Prior to 2002/1 the Part 1 examination was norm-referenced. All results presented here are calculated as the difference between a candidate's percentage mark and the particular pass mark for that diet, so that a score of zero indicates a candidate who has just achieved the pass mark, and a positive or negative score indicates the extent by which a candidate has passed or failed the examination.

### Background variables

The marks of UK graduates in the MRCP(UK) are known to relate to sex and to ethnicity [11]. In the main analysis, therefore, dummy variables at the candidate level were included for sex (male versus female) and for self-classified ethnicity (coded as white/non-white/not known). Date of birth, date of qualification and date of taking each part of the examination were known, and for Part 1, Part2 and PACES a variable was calculated indicating the time since qualifying as a doctor. Variables were also calculated for Part 2 and PACES giving the time elapsed since the first attempt at Part 1 and Part 2, respectively.

### Medical schools

Medical school descriptors in the MRCP(UK) database follow that used by the GMC and record only the university awarding the degree. As a result the five separate medical schools of the University of London are all recorded as 'London'.

### Compositional variables

As the MRCP(UK) database does not contain data at candidate level on a number of measures (for instance, pre-admission qualifications, such as A-levels or Highers), data from several different sources were aggregated to provide 'compositional variables'. About 90% of candidates in the main analysis qualified between 1999 and 2003 (Part 1: 96.4% of 7763; Part 2: 93.1% of 4470; PACES 89.6% of 4147), hence most would have entered medical school between 1994 and 1998, and so where possible compositional data were found for cohorts as close as possible to that entry period. We acknowledge that a proportion of students would have taken intercalated degrees or, for other reasons such as exam failure, would have qualified perhaps 6 or even 7 years after entry to a medical school. However, the MRCP(UK) database only contains information on date of qualification.

### Pre-admission qualifications

As mentioned above, results of A-levels and Highers for medical school entrants were not available. However, data on the pre-admission qualifications of medical school applicants receiving offers were obtained from the UCAS applicant cohorts of 1996 and 1997, which are in the public domain, and have been extensively analysed elsewhere [12]. Valid total point scores for A-levels and Highers (excluding general studies) were converted to *z*-scores for the entire population of applicants. Most applicants had only one of the two scores, which were used in the analysis, while for the minority of applicants with scores on A-levels and Highers, the higher of the two values was used as a measure of pre-admission qualifications. The UCAS database only included information on applicants receiving offers from particular medical schools, and aggregated means for individual medical schools were therefore calculated for all candidates receiving an offer at a particular school. It should be emphasized that since not all applicants receiving offers will subsequently enter a particular medical school, the correlation between average grades of applicants receiving offers and the average grades of entrants to a medical school will be less than perfect. Nevertheless the correlation is likely to be high.

### Perceptions of teaching quality

The 1991 cohort study, described elsewhere [13-15], asked medical students in their final year, in the years 1995–1997, to rate teaching on each individual basic medical science and clinical subject on four separate three-point scales of interest, difficulty, utility and time allocation. Here we consider only the measures for 'medicine' (i.e. hospital medicine/internal medicine). Aggregated means were based on the responses of 1486 students, with a median of 62 responses from each medical school (mean = 78; standard deviation (SD) = 85; SD range = 14–417).

### Career interest in hospital medicine

Students in the 1991 cohort study used a five-point scale to indicate their interest in 28 specialities, both at application to medical school and in the final year [16]. Mean scores, aggregated by medical school, were based on 2813 and 1472 respondents, with a median per school of 92 and 62, respectively.

### Proportion of graduates taking MRCP(UK) Part 1

Schools differ in the proportion of their graduates taking MRCP(UK). The number of graduates from each school known to have taken MRCP(UK) Part 1 at their first attempt during the eight diets from 2003 and 2005 was expressed as the percentage of the number of students from each school who registered provisionally with the GMC from 2001 to 2003 (data provided by CHMS; see Additional file 1). In Cambridge, Oxford and Edinburgh, 40%, 40% and 38% of graduates, respectively, took MRCP(UK), compared with 27%, 24% and 23% of graduates of Liverpool, Leicester and Birmingham, respectively.

### Performance at MRCGP

MRCGP (Membership of the Royal College of General Practitioners) is the principal postgraduate assessment for doctors in the UK wishing to become general practitioners. The percentage of graduates from each medical school who passed MRCGP at the first attempt between 1988 and 1991 is available from the study of Wakeford *et al*. [17]. Recent data for the period 2003–2006 are also available in the recent paper of Wakeford *et al*. [18].

### The Guardian analyses

The *Guardian *newspaper in the UK uses data provided by HESA (Higher Education Statistics Agency) and other sources to compile annual statistical analyses on students on individual courses at UK universities, which include an overall assessment of the course and a breakdown on a number of individual scores. The data and their nomenclature are confusing and our analysis here is restricted to those for 2003–2004 and 2005–2006.

### Statistical analysis

Conventional analyses were carried out using SPSS 13.0, multivariate multilevel statistical analyses were carried out with MLwiN 2.02 [19] and structural equation modelling used LISREL 8.54. In the main analysis, missing values for the three dependent variables were indicated in the data file, so that all available data could be included in the multilevel analysis [19,20]. Missing values for predictor variables were handled by mean substitution. The multilevel model had three levels, level1 for examination scores within candidates (which acts as a dummy), level2 for candidates and level3 for medical school of training. Covariance matrices were fitted separately at the candidate and medical school level. Residuals were extracted at the medical school level and tested for significance against the mean effect level of zero. The false discovery rate for each dependent variable was controlled using Benjamini and Liu's step-down method [21,22], which is broadly similar in its effect to the Bonferroni correction. Analysis of the compositional variables for the 19 medical schools was carried out in a separate analysis, with structural modelling of the correlation matrix performed using LISREL (see Additional file 1). The variables for each of the 19 schools in the LISREL analysis were treated as simple variables, with no attempt made to take into account differences in standard errors. For the additional analysis, only simple descriptive statistics were used.

## Results

### Main analysis

In the main analysis, results were available for 5827 candidates, 4040 of whom took Part 1, 3467 took Part 2 and 2888 took PACES for the first time, with 1248 taking all three parts within the time period, 2072 taking two parts and 2507 taking a single part.

### Multilevel modelling

#### Effect of background variables

The multilevel model examined scores in the three parts of the examination in a single model, simultaneously estimating both the variances of the scores and their covariances (using information from those candidates taking two or more parts). The main interest was in differences between medical schools. However, background variables were also significant. Males performed better at Part 1 and Part 2 (Part 1: *t *= 2.863, *p *= 0.0042; Part 2: *t *= 2.281, *p *= 0.0010) and females performed better at PACES (*t *= 5.777, *p *= 7.6 × 10^-9^); white candidates performed better at all three parts (Part1: *t *= 2.789, *p *= 0.0054; Part 2: *t *= 2.561, *p *= 0.010; PACES: *t *= 4.333, *p *= 0.000014). Candidates who had been qualified for a longer time performed significantly *worse *at Part 1 (*t *= 4.393, *p *= 0.000011), Part 2 (*t *= 32.1, *p *< 10^-12^) and PACES (*t *= 4.471, *p *= 0.000007); see Figures S1–3 in Additional file 1. Candidates who had a longer delay between Part 1 and Part 2 performed better at Part 2 (*t *= 8.175, *p *= 2 × 10^-16^), although there was no significant effect of the delay between Part 2 and PACES on PACES performance (*t *= 1.1, *p *= 0.271).

#### Correlations between examination parts

There were highly significant correlations at the candidate level for performance on first attempts at different parts of the examination (Part 1 with Part 2, *r *= 0.600, *n *= 2492, *p *< 0.001; Part 1 with PACES, *r *= 0.247, *n *= 1250, *p *< 0.001; Part 2 with PACES, *r *= 0.260, *n *= 2074, *p *< 0.001; see Figures S4–6 in Additional file 1) indicating a reasonably high degree of stability in performance within individuals.

#### Medical school effects

Multilevel modelling found a highly significant overall effect of medical schools (χ^2 ^= 300.57, six degrees of freedom, *p *< 0.001), with significant variance between schools for Part 1 (variance = 8.345, standard error (SE) = 2.85, *t *= 2.928, *p *= 0.0034), Part 2 (variance = 2.557, SE = 0.916, *t *= 2.791, *p *= 0.0053) and PACES (variance = 0.787, SE = 0.327, *t *= 2.401, *p *= 0.016). (The full fitted multilevel model is shown in Figures S7 and S8 in Additional file 1). Medical school variance is greatest for Part 1 and least for PACES, but those differences also reflect differences in total variance (82.6 for Part 1, 40.4 for Part 2 and 28.4 for PACES). The coefficient of variation, expressed as medical school SD as a percentage of total SD, is 31.7% for Part 1, 25.1% for Part 2 and 16.6% for PACES.

**Figure 1:**
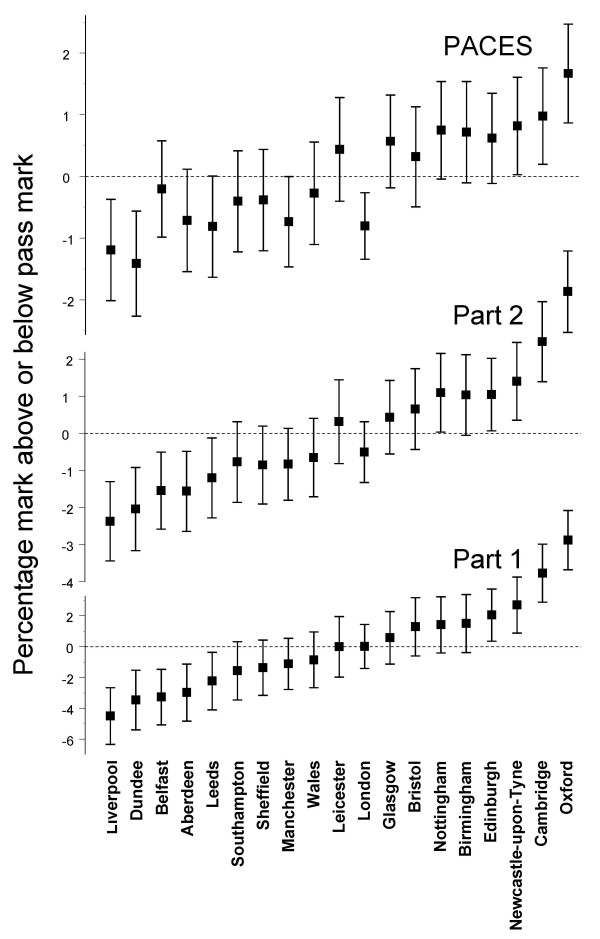
**Medical school effects for the Part 1, Part 2 and PACES exams of MRCP(UK)**. For all three parts of the examination, medical schools are sorted by the size of the effect at the Part 1 examination. Error bars indicate ± 1 SD. Note that absolute values are different for the three examinations (see the text).

Figure 1 shows the residuals for each part, rank ordered in each case by the effect found in Part 1 of the examination. In Part 1 of the examination and following correction for multiple testing, the graduates of Oxford, Cambridge and Newcastle-upon-Tyne performed significantly better than average, and the graduates of Liverpool, Dundee, Belfast and Aberdeen performed significantly worse than average.

At the medical school level, performance at Part 2 correlated significantly with performance at Part 1 (*r *= 0.981, *p *= 0.004), with the same schools as for Part 1 showing significant differences from the mean.

In the PACES examination, the correlation with performance at Part 1 and Part 2 was a little lower than that found between Part 1 and Part 2, but was also highly significant (Part 1 with PACES: *r *= 0.849, *p *= 0.0114; Part 2 with PACES: *r *= 0.897, *p *= 0.0096). Four schools performed significantly differently from average, three of which were also significant at Part 1 and Part 2 (Oxford above average, and Dundee and Liverpool below average) and in addition London also performed significantly worse than average, although London graduates had been almost precisely at the average for Parts 1 and 2. (Scattergrams of the relationship between medical school effects at Part 1, Part 2 and PACES can be found in Figure S9 in Additional file 1).

### Analysis of compositional variables

In this section we analyse data at the level of the 19 medical schools, and whenever phrases such as 'higher pre-admission qualifications' are used it must be emphasized that this refers to 'medical schools whose candidates have higher pre-admission qualifications' and does *not *mean 'individual candidates with higher pre-admission qualifications'. Correlations and structural models at the individual and school level may be similar but they need not be [20], and the analyses described here are specifically at the school level of analysis.

**Figure 2:**
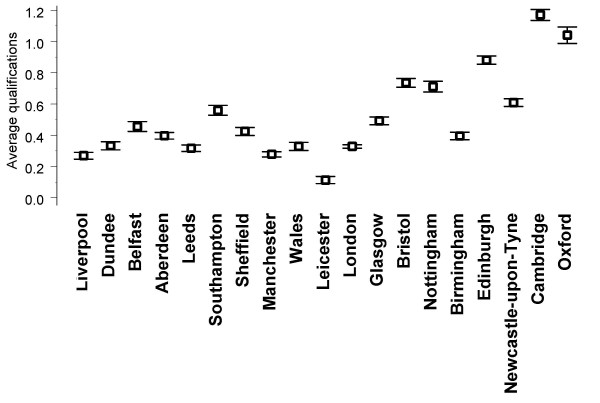
**Average pre-admission qualifications of applicants receiving offers at UK medical schools**. Values are *z*-scores of A-levels and Highers and are standardized across all applicants (and hence those receiving offers tend to have above average values). Error bars indicate ± 1 SE and because sample sizes are large (typically of the order of 500 and over 5000 in the case of London), error terms are small (see the text).

Table 1 shows correlations between a school's average performance in the three parts of the exam and the compositional variables describing the school (for more details see Additional file 1). The highest correlations with MRCP(UK) performance are with pre-admission qualifications (see Figure 2), the correlation between aggregated mean pre-admission qualification and aggregated mean performance at Part 1 being highly significant (*r *= 0.779, *n *= 19, *p *< 0.001) and remaining significant when Oxford and Cambridge are omitted from the analysis (*r *= 0.566, *n *= 17, *p *= 0.018).

**Table 1:** Correlations of medical school performance and compositional variables

	Part 1	Part 2	PACES
Mean pre-admission qualifications at A-levels or Highers	**0.779** ***p *= 0.000085**	**0.773** ***p *= 0.000011**	**0.704** ***p *= 0.00076**
Interest in career in hospital medicine at application	0.205 *p *= 0.401	0.196 *p *= 0.421	0.223 *p *= 0.358
Interest in career in hospital medicine in final year	**0.510** ***p *= 0.026**	**0.522** ***p *= 0.022**	**0.500** ***p *= 0.029**
Interest of teaching in general medicine	**0.588** ***p *= 0.0081**	**0.568** ***p *= 0.011**	**0.483** ***p *= 0.036**
Difficulty of teaching in general medicine	0.128 *p *= 0.603	0.143 *p *= 0.559	0.153 *p *= 0.532
Usefulness of teaching in general medicine	0.223 *p *= 0.358	0.234 *p *= 0.334	0.219 *p *= 0.369
More time needed for teaching of general medicine	0.023 *p *= 0.926	0.009 *p *= 0.972	-0.049 *p *= 0.842
Percentage of graduates taking MRCP(UK)	**0.613** ***p *= 0.005**	**0.575** ***p *= 0.010**	**0.478** ***p *= 0.038**
Pass rate at MRCGP, 1988–1991	**0.601** ***p *= 0.0065**	**0.611** ***p *= 0.0054**	**0.532** ***p *= 0.019**
Pass rate at MRCGP, 2003–2006	**0.690** ***p *= 0.0011**	**0.726** ***p *= 0.0004**	**0.792** ***p *= 0.00005**

Correlations at the medical school level (*n *= 19) between performance at Part 1, Part 2 and PACES and compositional variables describing the medical schools. Values in bold are significant with *p *< 0.05.

Although medical schools with a higher proportion of graduates taking MRCP(UK) tended to have higher pre-admission qualifications (*r *= 0.833, *p *= 0.001, *n *= 19), there was a weaker correlation between a medical school's performance at MRCP(UK) and the proportion of its graduates taking the exam (*r *= 0.613, *p *= 0.005, *n *= 19). The proportion of graduates taking MRCP(UK) did not predict outcome after pre-admission qualifications were taken into account (β = -0.175, *p *= 0.559), whereas pre-admission qualifications did predict outcome after taking into account the proportion of graduates taking MRCP(UK) (β = 0.928, *p *= 0.006). There is therefore no independent effect of the proportion of a school's graduates taking MRCP(UK).

**Figure 3:**
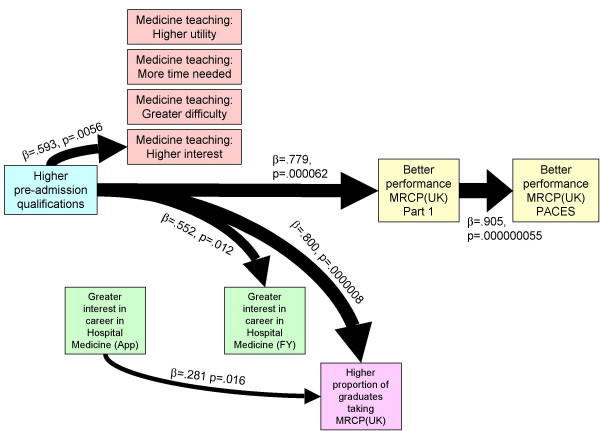
**Structural equation model for the causal relationship between the variables at the medical school level**. Path strengths are shown as ∃ (standardized) path coefficients and significance levels based on a *t*-statistic with 17 degrees of freedom. The width of paths is proportional to the path coefficient. The saturated model allowed all variables to the left of a variable to have a causal influence on that variable and non-significant paths were removed until paths remaining were significant with *p *< 0.05. Paths not shown as causal arrows did not reach significance with *p *< 0.05.

The relationship between all of the variables and Part 1 performance was examined using multiple regression, and only pre-admission qualifications predicted performance at MRCP(UK). The structural equation model in Figure 3 shows that the only variable with a direct or indirect effect on Part 1 or PACES performance is higher pre-admission qualifications, which had separate effects with teaching being rated as more interesting, a greater career interest in medicine in the final year and a higher proportion of graduates taking MRCP(UK).

Of particular theoretical interest (see the discussion) is that the performance of a medical school's graduates at MRCP(UK) correlated highly with performance in the MRCGP when taken in 2003–2005 and a little less so with the performance in 1988–1991 (see Table 1).

#### Performance in relation to the Guardian assessments

Table 2 shows correlations between the variables reported in the two compilations of data by the *Guardian*, and outcome at Part 1, Part 2 and PACES. The highest correlations, for both sets of data, are with the entry scores, which are based on university admission criteria. Using a forward entry multiple regression, in which the entry score based on the 2003–2004 data was entered first, no other variables apart from university admission criteria were significant predictors of Part 1, Part 2 or PACES performance.

**Table 2:** Correlations of medical school performance and *Guardian *scores.

Years data are mainly based on	*Guardian *scores	Part 1	Part 2	PACES
2005–2006	Overall score	**0.578** ***p *= 0.010**	**0.558** ***p *= 0.013**	0.398 *p *= 0.092
	Teaching score (based on National Student Survey) (Note:*N *= 14)	0.218 *p *= 0.454	0.242 *p *= 0.405	0.262 *p *= 0.366
	Feedback score (based on National Student Survey) (Note: *N *= 14)	-0.101 *p *= 0.731	-0.048 *p *= 0.871	0.088 *p *= 0.765
	Spending per student	0.414 *p *= 0.078	0.363 *p *= 0.127	0.131 *p *= 0.594
	Staff/student ratio	0.001 *p *= 0.998	-0.108 *p *= 0.941	-0.077 *p *= 0.755
	Entry score (based on UCAS tariff scores)	**0.603** ***p *= 0.006**	**0.612** ***p *= 0.005**	**0.572** ***p *= 0.011**
2003–2004	Overall score	**0.540** ***p *= 0.017**	**0.486** ***p *= 0.035**	0.308 *p *= 0.200
	Staff score	0.118 *p *= 0.630	0.081 *p *= 0.741	0.035 *p *= 0.888
	Spending per student	**0.499** ***p *= 0.030**	0.445 *p *= 0.056	0.239 *p *= 0.325
	Staff/student ratio	0.223 *p *= 0.358	0.191 *p *= 0.434	0.128 *p *= 0.601
	Entry score (based on UCAS tariff scores)	**0.561** ***p *= 0.013**	**0.590** ***p *= 0.008**	**0.581** ***p *= 0.009**

Correlations at the medical school level between performance at Part 1, Part 2 and PACES and the variables derived by the *Guardian *from HESA and other statistics (see the text). Values in bold are significant with *p *< 0.05. *N *= 19 for most variables, except for those based on the National Student Survey, which has *N *= 14 owing to either to low numbers of respondents or differences between UK regions.

### Additional analysis

**Figure 4:**
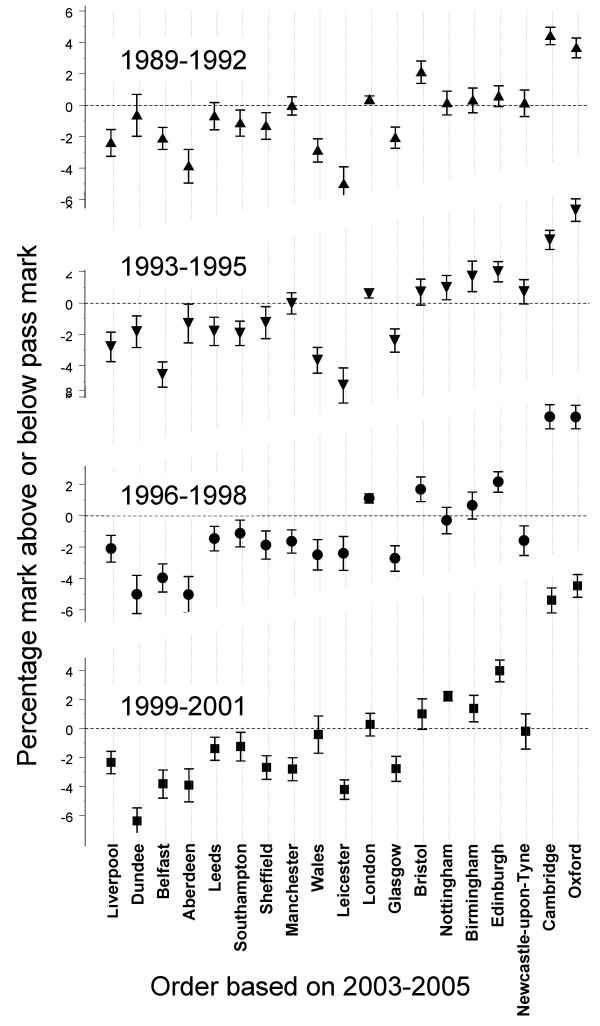
**Differences between medical schools for candidates taking Part 1 MRCP(UK) from 1989 to 2001**. Medical schools are sorted by Part 1 performance in 2003–2005. Error bars indicate ± 1 SE.

Data were available for a total of 22453 graduates of UK medical schools taking the Part 1 exam for the first time in the 51 diets from 1989/1 to 2005/3. Figure 4 shows performance of candidates from different schools taking Part 1 in 1989–1992, 1993–1995, 1996–1998 and 1999–2001, with schools plotted in the order found in the main analysis for 2003–2005. Performance in 2003–2005 correlated 0.739 with performance in 1989–1992, 0.816 with performance in 1993–1995, 0.848 with performance in 1996–1998 and 0.884 with performance in 1999–2001 (*n *= 19, *p *< 0.001 for all correlations). Excluding Oxford and Cambridge, the correlations were 0.493, 0.618, 0.654 and 0.723, respectively (*n *= 17, *p *= 0.045, 0.008, 0.004 and 0.001). Detailed, year-by-year graphs of trends within individual schools can be found in Figures S10 and S11a-11e in Additional file 1.

## Discussion

Our analysis shows that candidates who have trained at different UK medical schools perform differently in the MRCP(UK) examination. In 2003–2005, 91%, 76% and 67% of students from Oxford, Cambridge and Newcastle passed Part 1 at their first attempt, compared with 32%, 38%, 37% and 41% of Liverpool, Dundee, Belfast and Aberdeen graduates, so that, for instance, twice as many Newcastle graduates pass the exam first time compared with Liverpool graduates (odds ratio = 4.3×).

At the medical school level, performance at Part 1 correlates almost perfectly with performance at Part 2 (and both are multiple-choice examinations), while performance at PACES, which is a clinical examination, still correlates highly with Parts 1 and 2, although there are some small changes in rank order, the most notable being that London graduates perform worse than average at PACES but not at Part 1 and Part 2.

School-leaving examinations are known at the individual level to predict performance in undergraduate medical examinations and in postgraduate careers [23,24]. Although pre-admission academic qualifications correlate significantly with MRCP(UK) Part 1 performance at the medical school level (*r *= 0.779), that correlation is substantially less than the correlation found between Part 1 and Part 2 of the examination (*r *= 0.992). Pre-admission qualifications therefore account for about 62% of the accountable variance, leaving about 38% of the school-level variance dependent on other, unknown, factors. It should be emphasized that because sex and ethnic origin have been entered into the multilevel model at an individual level, there can be no differences at medical school level attributable to ethnicity or sex.

There are at least three broad types of explanation for the differences we have found: differences in those entering the schools (selection effects); differences in education or training at the school (training effects); or differences owing to students from different schools preferring different postgraduate careers (career preference effects).

Selection effects would predict that better qualified students enter schools such as Oxford, Cambridge and Newcastle-upon-Tyne (and Oxford and Cambridge, in particular, have traditionally demanded very high A-levels), so that the better-qualified entrants to those schools would also be likely to perform better in postgraduate examinations. At the individual level it is known that A-level results correlate with performance in MRCP(UK) Part 1 [24] and there are also clear differences in the average pre-admission qualifications of applicants receiving offers at different medical schools (see Figure 2). Our analysis of compositional variables leaves little doubt that one-half or more of the variance between schools can be explained by differences in intake, and that is supported by the correlations found with the data reported in the *Guardian *tables, which are compiled from a range of official statistics (Table 2). However, even at Part 1 the correlation leaves at least one-third of the variance unexplained. In particular, MRCP(UK) performance is about one SD higher than predicted from pre-admission qualifications alone for Leicester, Oxford, Birmingham, Newcastle-upon-Tyne and London, and about one SD lower than expected for Southampton, Dundee, Aberdeen, Liverpool and Belfast. Neither can differences in pre-admission qualifications explain the relative underperformance of London graduates at PACES, compared with Part 1 and Part 2. Pre-admission qualifications are a part of the story, but are not the entire explanation of medical school differences and the remaining variance is most likely to be related either to other differences in the intake of schools or to differences in the education provided by those schools.

Career preference effects would occur if the differential performance of graduates on MRCP(UK) reflects a form of self-selection into different specialities (and Parkhouse reported, for instance, that amongst those qualifying between 1974 and 1983 that hospital medicine was particularly popular for Oxford, London and Wales graduates, and particularly unpopular for Aberdeen, Dundee and Leicester graduates [25]). If popularity also equated to status and kudos, then it might be that the most academically gifted students at one school might prefer to go into one particular speciality, whereas at another school they might prefer a different speciality. Candidates would then perform better if they came from schools where a higher proportion of graduates took the MRCP(UK). However, our data show that not to be the case, as the correlation of performance and the proportion taking the exam was non-significant after pre-admission qualifications are taken into account.

Career preference effects also predict that if training at all schools is on aggregate equivalent, then schools performing better at one particular postgraduate examination, because their better students prefer to take it, should also perform *less *well at other examinations which are taken by their less gifted graduates. Overall there would then be a *negative *correlation in the ordering of schools across any pair of postgraduate examinations. In a study of performance at MRCGP in the early 1990s [17], graduates of Oxford, Cambridge and Newcastle-upon-Tyne ranked 1st, 5th and 7th in performance, compared with Belfast, Aberdeen, Dundee and Liverpool graduates who ranked 16th, 23rd, 24th and 26th out of the 27 UK medical schools, with an overall *positive *correlation of effect sizes of *r *= 0.480 (*p *= 0.038, *n *= 19). More recent data for the MRCGP from 2003–2006 show a similar and somewhat stronger trend (see Table 1). Such *positive *correlations, if confirmed by other examinations, would make the career selection explanation unlikely.

Institutions can differ in the amount of 'value' that they add, an effect well known in secondary education [26]. Training effects would predict that teaching and training in general medicine at some schools is a better preparation for MRCP(UK) than at others, perhaps because of differences in course emphasis or focus, so that candidates subsequently perform better at the MRCP(UK). If career preferences and pre-admission qualifications cannot explain all of the differences between medical schools, then a reasonable conclusion is that that medical schools also differ in the quality of their training in general medicine. Some schools may therefore be adding more value to their students than others, in relation to taking the MRCP(UK), even taking into account differences in pre-admission qualifications. However, it is of interest that none of the teaching-related measures in the *Guardian *compilations correlate with MRCP(UK) performance.

The MRCP(UK) examinations are typically taken early in the career, The impact of university teaching on performance is supported by our finding that recency of graduation is a predictor of performance in all three parts of the examination. The coefficient of variation for medical school differences was largest for Part 1 and smallest for PACES, suggesting that postgraduate education dilutes the effects of undergraduate training as time passes. Understanding the mechanisms by which medical school teaching might affect postgraduate examination performance requires more background information than we have available. It is interesting that when a university's students are more likely to report that the teaching of medicine is 'very interesting', then graduates subsequently perform better at MRCP(UK). However, that effect does seem to be secondary to pre-admission qualifications, with students from schools with higher pre-admission qualifications also reporting the teaching of medicine to be more interesting. Teaching can be affected not only by the activities of teachers and students, but also by the environment and institutions in which teaching occurs. A case of particular interest is London, the only university for which there is a specific underperformance of graduates on PACES, the clinical examination of MRCP(UK), and London's medical schools have undergone repeated reorganizations over the past two decades, which might in part explain the effects on clinical teaching. As the data are aggregated for all London schools, this is difficult to explore further here. An additional confounding issue for all schools of medicine is the constant change in curricula. However, our additional analysis of Part1 data going back to those taking the exam in 1989 (who would have entered medical school in about 1982) shows that the broad pattern of results we have found is long-standing, and therefore could only partly be explained by the changes in medical education initiated by the GMC in *Tomorrow's Doctors *in 1993 [27]. A detailed examination of individual medical schools (see Figures S11a-11e in additional file 1) shows that for many schools there has been little variation in relative performance between 1989 and 2005. Problem-based learning, introduced in Glasgow, Liverpool and Manchester, has had little obvious impact in the latter two schools, although performance did increase in Glasgow. Despite many, much criticised reorganizations in London, performance overall has improved. Oxford and Cambridge both showed sudden increases in performance in the late 1990s, as did Wales. Other schools showed fluctuations, but the overwhelming impression is of constancy rather than change, suggesting that curricular and other changes have had little impact on relative performance of schools.

The MRCP(UK) consists of both written and clinical examinations, and detailed analyses of its rationale and behaviour have been presented elsewhere [3-8]. Of course, the examination does not assess the entire range of knowledge, skills and attitudes necessary to be a successful physician, although it does cover diagnosis and management within internal medicine comprehensively, and the PACES examination assesses a wide range of practical skills, including physical examination, recognition of signs, management of patients, history-taking, communication with patients and relatives, and handling difficult ethical situations. Current work suggests that PACES, in particular, assesses all of the competencies that *Modernising Medical Careers *recognizes should be assessed in such an examination, and it is an important, coherent and central part of the assessment of competencies within the UK that the GMC and PMETB recognize as needing to be assessed. However, MRCP(UK) cannot assess all of the necessary competencies and it is possible that some of those not assessed are also inculcated better by some medical schools than others, and this possibility must await further evidence from other sources.

## Conclusion

The Tooke Report of October 2007 [2] stated that British medical education urgently needed,

" ... answers to some fundamental questions. How does an individual student from one institution compare with another from a different institution? Where should that student be ranked nationally? Are there any predictors for later careers choices and are these evident in undergraduate training? Which medical schools' students are best prepared for the Foundation Years and, crucially, what makes the difference?" ([2], p. 127)

The earlier GMC report of June 2006, *Strategic Options for Undergraduate Medical Education *[1], had also included a discussion on the potential need to introduce a national medical assessment to ensure that all UK medical graduates have attained an agreed minimum standard of competence. However, the report also highlighted the very limited evidence that existed to support the contention that significant differences in ability existed between graduates of different UK universities. However, an absence of evidence is not evidence of absence, and there are many reasons to believe that schools might differ [28]; a study in the US, for instance, found that graduates of different medical schools differed in their likelihood of malpractice claims [29]. We believe that our data provide a *prima facie *case that differences in performance exist between UK medical schools, and thus support the case for the routine collection and audit of performance data of UK medical graduates at all postgraduate examinations, as well as the introduction of a national licensing examination.

## Competing interests

ICM, AE and JD are Fellows of one or more of the three colleges that administer the MRCP(UK), JD is Academic Vice-President of the Royal College of Physicians of London and AE is Registrar of the Royal College of Physicians of Edinburgh. JM and LC are or were paid employees of the MRCP(UK) Central Office. AdC acts as a consultant to the MRCP(UK) through the NBME-MRCP(UK) consulting agreement and ICM is educational advisor to the MRCP(UK). ICM, AE, JD and AdC sit on various committees administering the MRCP(UK), and all receive expenses for travelling to meetings, etc., but are otherwise not reimbursed. JD, AE and ICM work and have worked in a number of medical schools, and JD is Vice Dean of the medical school of University College London. Publication costs of this paper have been met by the MRCP(UK) Central Office.

## Authors' contributions

The idea for the study was developed jointly by ICM, AE and AdC, data preparation was carried out by JM and LC, statistical modelling was performed by ICM and AdC, various drafts of the paper were written and edited by AdC, AE and ICM, and all authors have revised and approved the final manuscript.

## Pre-publication history

The pre-publication history for this paper can be accessed here:



## Supplementary Material

Additional file 1 Supplementary Information. Additional statistical analyses and graphs for individual medical schools. Click here for file
