# Supplementary material for: Graduates of different UK medical schools show substantial differences in performance on MRCP(UK) Part 1, Part 2 and PACES examinations
Source: BMC Med. 2008 Feb 14;6:5. doi: 10.1186/1741-7015-6-5 (PMC2265293; doi:10.1186/1741-7015-6-5)

Supplementary Information

1. Numbers of graduates from UK medical schools

2. Structural equation modelling

3. Graphs for individual medical schools by year that graduates took MRCP(UK) Part 1

Supplementary figures 1 to 10 and 11a to 11e.

1. *Numbers of graduates from UK medical schools*. The table below shows the number of graduates of UK medical schools who took MRCP(UK) Part 1 for the first time in the diets of 2003/2 to 2005/3. Since most UK graduates tend to take MRCP(UK) soon after the regulations allow, which is eighteen months after qualification, a typical graduate in June 2001 would be able to take the exam early in 2003. The first data column (*a*) shows the number of graduates from each school who provisionally registered with the GMC in 2001 to 2003 (i.e. broadly equivalent to the eight diets we have analysed), and the second column (*b*) the numbers taking MRCP(UK) for the first time in the 2003/2 to 2005/3 diets. Column *c* shows *a* expressed as a percentage of *b*, and column *d* shows *c* relative to the overall percentage (final row of column *c*).

	Number of graduates provisionally registering with GMC in 2001-2003	Number taking MRCP(UK) Pt 1 2003-2005	100 x *a* /*b*	Ratio *c*/30.21
	(a)	*(b)*	*(c)*	*(d)*
Aberdeen	146	499	29.3	0.969
Belfast	161	500	32.2	1.066
Birmingham	135	581	23.2	0.769
Bristol	130	429	30.3	1.003
Cambridge	133	330	40.3	1.334
Dundee	116	422	27.5	0.910
Edinburgh	233	610	38.2	1.264
Glasgow	220	713	30.9	1.021
Leeds	139	503	27.6	0.915
Leicester	112	471	23.8	0.787
Liverpool	146	543	26.9	0.890
London	1213	3852	31.5	1.042
Manchester (inc St Andrews)	258	937	27.5	0.912
Newcastle-Upon-Tyne	160	546	29.3	0.970
Nottingham	157	544	28.9	0.955
Oxford	121	306	39.5	1.309
Sheffield	171	612	27.9	0.925
Southampton	128	443	28.9	0.957
UWCM	161	533	30.2	1.000
**Total**	**4040**	**13374**	**30.2**	**1**

2. *Structural equation modelling.* Structural equation modelling used LISREL. Selected output from the program, including the commands and the correlation matrix, is shown below. The saturated model allowed all variables to the left of a variable to have a causal influence on it (via the BETA matrix), with the exception that the four measures of medicine teaching (MEDINT MEDDIF MEDUSE MEDTIME) related to one another through the PSI matrix which was saturated for those relationships. The saturated model was fitted initially, and least significant paths removed sequentially until t>2 for all paths remaining.

```
DA NI=11 NO=19 ma=km
km fu
*
 1.000  .177  .518  .160  .225 -.017  .552  .850  .779  .773  .704
  .177 1.000  .119  .019  .055  .176 -.022  .423  .205  .196  .223
  .518  .119 1.000  .115 -.192  .240  .470  .478  .588  .568  .483
  .160  .019  .115 1.000 -.157  .364 -.111  .074  .128  .143  .153
  .225  .055 -.192 -.157 1.000 -.739 -.118  .141  .223  .234  .219
-.017  .176  .240  .364 -.739 1.000  .009  .085  .023  .009 -.049
  .552 -.022  .470 -.111 -.118  .009 1.000  .545  .510  .522  .500
  .850  .423  .478  .074  .141  .085  .545 1.000  .613  .575  .478
  .779  .205  .588  .128  .223  .023  .510  .613 1.000  .992  .905
  .773  .196  .568  .143  .234  .009  .522  .575  .992 1.000  .945
  .704  .223  .483  .153  .219 -.049  .500  .478  .905  .945 1.000

la
*
qual medapp medint meddif meduse medtime medfy Ptake Part1 Part2 PACES
se
qual medapp medint meddif meduse medtime medfy Ptake Part1 PACES/
mo ny=10 te=di,fr be=sd ps=sy,fr
pa be
*
0 0   0 0 0 0 0 0 0   0
0 0   0 0 0 0 0 0 0   0
1 0   0 0 0 0 0 0 0   0
0 0   0 0 0 0 0 0 0   0
0 0   0 0 0 0 0 0 0   0
0 0   0 0 0 0 0 0 0   0
1 0   0 0 0 0 0 0 0   0
1 1   0 0 0 0 0 0 0   0
1 0   1 0 0 0 0 0 0   0
0 0   0 0 0 0 0 0 1   0

pa ps
*
1
0 1
0 0 1
0 0 1 1
0 0 1 1 1
0 0 1 1 1 1
0 0 0 0 0 0 1
0 0 0 0 0 0 0 1
0 0 0 0 0 0 0 0 1
0 0 0 0 0 0 0 0 0 1
OU me=ml MI RS EF MR SS SC  nd=3 it=1000


                          Number of Input Variables 11
                          Number of Y - Variables   10
                          Number of X - Variables    0
                          Number of ETA - Variables 10
                          Number of KSI - Variables  0
                          Number of Observations    19
```

```
Number of Iterations =  5

 LISREL Estimates (Maximum Likelihood)

         BETA

               qual     medapp     medint     meddif     meduse    medtime
           --------   --------   --------   --------   --------   --------
     qual      - -        - -        - -        - -        - -        - -
   medapp      - -        - -        - -        - -        - -        - -
   medint     0.593       - -        - -        - -        - -        - -
            (0.187)
              3.170
   meddif      - -        - -        - -        - -        - -        - -
   meduse      - -        - -        - -        - -        - -        - -
  medtime      - -        - -        - -        - -        - -        - -
    medfy     0.552       - -        - -        - -        - -        - -
            (0.197)
              2.809
    Ptake     0.800      0.281       - -        - -        - -        - -
            (0.106)    (0.106)
              7.576      2.664
    Part1     0.779       - -        - -        - -        - -        - -
            (0.148)
              5.271
    PACES      - -        - -        - -        - -        - -        - -

        BETA
              medfy      Ptake      Part1      PACES
           --------   --------   --------   --------
     qual      - -        - -        - -        - -
   medapp      - -        - -        - -        - -
   medint      - -        - -        - -        - -
   meddif      - -        - -        - -        - -
   meduse      - -        - -        - -        - -
  medtime      - -        - -        - -        - -
    medfy      - -        - -        - -        - -
    Ptake      - -        - -        - -        - -
    Part1      - -        - -        - -        - -
    PACES      - -        - -       0.905       - -
                                  (0.100)
                                    9.026

         PSI
               qual     medapp     medint     meddif     meduse    medtime
           --------   --------   --------   --------   --------   --------
     qual     1.000
            (0.333)
              3.000
   medapp      - -       1.000
                       (0.333)
                         3.000
   medint      - -        - -       0.737
                                  (0.246)
                                    3.000
   meddif      - -        - -       0.020      1.000
                                  (0.202)    (0.333)
                                    0.099      3.000
   meduse      - -        - -      -0.325     -0.157      1.000
                                  (0.216)    (0.239)    (0.333)
                                   -1.504     -0.658      3.000
  medtime      - -        - -       0.250      0.364     -0.739      1.000
                                  (0.211)    (0.251)    (0.293)    (0.333)
                                    1.186      1.451     -2.521      3.000
    medfy      - -        - -        - -        - -        - -        - -
    Ptake      - -        - -        - -        - -        - -        - -
    Part1      - -        - -        - -        - -        - -        - -
    PACES      - -        - -        - -        - -        - -        - -

         PSI

              medfy      Ptake      Part1      PACES
           --------   --------   --------   --------
    medfy     0.695
            (0.232)
              3.000
    Ptake      - -       0.201
                       (0.067)
                         3.000
    Part1      - -        - -       0.393
                                  (0.131)
                                    3.000
    PACES      - -        - -        - -       0.181
                                             (0.060)
                                               3.000
```

```
                    Goodness of Fit Statistics

                      Degrees of Freedom = 33
          Minimum Fit Function Chi-Square = 28.377 (P = 0.697)
  Normal Theory Weighted Least Squares Chi-Square = 22.447 (P = 0.917)
             Estimated Non-centrality Parameter (NCP) = 0.0
         90 Percent Confidence Interval for NCP = (0.0 ; 2.463)

                  Minimum Fit Function Value = 1.577
            Population Discrepancy Function Value (F0) = 0.0
         90 Percent Confidence Interval for F0 = (0.0 ; 0.137)
         Root Mean Square Error of Approximation (RMSEA) = 0.0
       90 Percent Confidence Interval for RMSEA = (0.0 ; 0.0644)
          P-Value for Test of Close Fit (RMSEA < 0.05) = 0.939

             Expected Cross-Validation Index (ECVI) = 4.278
       90 Percent Confidence Interval for ECVI = (4.278 ; 4.415)
                    ECVI for Saturated Model = 6.111
                  ECVI for Independence Model = 8.206

 Chi-Square for Independence Model with 45 Degrees of Freedom = 127.703
                      Independence AIC = 147.703
                          Model AIC = 66.447
                        Saturated AIC = 110.000
                      Independence CAIC = 167.147
                          Model CAIC = 109.224
                        Saturated CAIC = 216.944

                     Normed Fit Index (NFI) = 0.778
                  Non-Normed Fit Index (NNFI) = 1.076
               Parsimony Normed Fit Index (PNFI) = 0.570
                  Comparative Fit Index (CFI) = 1.000
                  Incremental Fit Index (IFI) = 1.049
                    Relative Fit Index (RFI) = 0.697

                        Critical N (CN) = 35.745


              Root Mean Square Residual (RMR) = 0.104
                        Standardized RMR = 0.104
                  Goodness of Fit Index (GFI) = 0.800
            Adjusted Goodness of Fit Index (AGFI) = 0.667
            Parsimony Goodness of Fit Index (PGFI) = 0.480
```

3. Graphs for individual medical schools by year that graduates took MRCP(UK) Part 1.

Figure 4 (main text) gives an impression of the stability and change in the performance of medical schools for those taking the examination for the first time over the period 1989 to 2005 (i.e. who typically graduated between 1987 and 2003, and therefore typically would have entered medical school between about 1983 and 1997). However it is useful also to plot graphs, year by year, for individual medical schools. A complication in so doing is shown in Supplementary figure 10, which plots the mean score of all UK graduates at their first attempt by year, the score, as previously, being plotted relative to the pass mark for each diet. It is clear that although mean performance was stable over the period 1989 to about 1998, performance then began to rise, for reasons which are not entirely clear. The result is that while a majority of UK candidates would fail the exam at their first attempt in 1989, a small majority is now passing the exam at the first attempt. Whatever the reasons for that change, it makes it somewhat complicated to visualise the *relative* performance of candidates from individual schools, when they are plotted in the same way, since the trend of Supplementary Figure 10 has to be taken into account. As a result, for the graphs of performance of individual medical schools shown in Supplementary Figure 11, we have subtracted the overall mean scores for each year shown in Supplementary Figure 10, so that all changes should be interpreted as the performance of candidates at a particular medical school relative to the performance of all UK candidates.

Correlations across years. An important question concerns the stability of the ordering of medical schools across years. A correlation matrix was therefore generated showing the correlation of the mean score in each year with the mean score in each other year (see below). The stability across an interval of N years was then calculated as the mean of the correlations separated by that number of years (for which there were 16 correlations separated by 1 year, 15 separated by 2 years, through to 1 correlation separated by 16 years).

Correlations of medical school scores across different numbers of years.

	**1989**	**1990**	**1991**	**1992**	**1993**	**1994**	**1995**	**1996**	**1997**	**1998**	**1999**	**2000**	**2001**	**2002**	**2003**	**2004**	**2005**
**1989**	1	0.713	0.551	0.509	0.588	0.450	0.559	0.708	0.719	0.548	0.547	0.524	0.477	0.613	0.434	0.520	0.582
**1990**	0.713	1	0.767	0.669	0.797	0.667	0.741	0.857	0.764	0.870	0.831	0.818	0.717	0.772	0.678	0.508	0.749
**1991**	0.551	0.767	1	0.723	0.691	0.657	0.799	0.745	0.602	0.752	0.740	0.627	0.610	0.505	0.572	0.390	0.659
**1992**	0.509	0.669	0.723	1	0.777	0.804	0.767	0.607	0.559	0.679	0.677	0.663	0.612	0.625	0.622	0.666	0.645
**1993**	0.588	0.797	0.691	0.777	1	0.602	0.667	0.704	0.524	0.634	0.582	0.730	0.489	0.744	0.648	0.508	0.571
**1994**	0.450	0.667	0.657	0.804	0.602	1	0.840	0.681	0.635	0.641	0.772	0.745	0.721	0.641	0.674	0.681	0.736
**1995**	0.559	0.741	0.799	0.767	0.667	0.840	1	0.786	0.714	0.763	0.794	0.828	0.789	0.711	0.804	0.714	0.759
**1996**	0.708	0.857	0.745	0.607	0.704	0.681	0.786	1	0.851	0.731	0.746	0.789	0.756	0.777	0.753	0.629	0.781
**1997**	0.719	0.764	0.602	0.559	0.524	0.635	0.714	0.851	1	0.776	0.822	0.760	0.840	0.775	0.693	0.696	0.840
**1998**	0.548	0.870	0.752	0.679	0.634	0.641	0.763	0.731	0.776	1	0.936	0.783	0.825	0.780	0.652	0.614	0.835
**1999**	0.547	0.831	0.740	0.677	0.582	0.772	0.794	0.746	0.822	0.936	1	0.766	0.828	0.764	0.650	0.612	0.883
**2000**	0.524	0.818	0.627	0.663	0.730	0.745	0.828	0.789	0.760	0.783	0.766	1	0.882	0.863	0.820	0.675	0.743
**2001**	0.477	0.717	0.610	0.612	0.489	0.721	0.789	0.756	0.840	0.825	0.828	0.882	1	0.797	0.789	0.753	0.803
**2002**	0.613	0.772	0.505	0.625	0.744	0.641	0.711	0.777	0.775	0.780	0.764	0.863	0.797	1	0.783	0.797	0.848
**2003**	0.434	0.678	0.572	0.622	0.648	0.674	0.804	0.753	0.693	0.652	0.650	0.820	0.789	0.783	1	0.773	0.782
**2004**	0.520	0.508	0.390	0.666	0.508	0.681	0.714	0.629	0.696	0.614	0.612	0.675	0.753	0.797	0.773	1	0.792
**2005**	0.582	0.749	0.659	0.645	0.571	0.736	0.759	0.781	0.840	0.835	0.883	0.743	0.803	0.848	0.782	0.792	1

The mean correlations lagged from one to sixteen years were 0.785, 0.744, 0.739, 0.704, 0.689, 0.707, 0.734, 0.710, 0.691, 0.669, 0.601, 0.611, 0.581, 0.533, 0.634, and 0.581.

Supplementary Figures (see following pages for figures).

a. *Supplementary figure 1*: Part 1 mark in relation to time in years since qualifying.

b. *Supplementary figure 2*: Part 2 mark in relation to time in years since qualifying.

c. *Supplementary figure 3*: PACES mark in relation to time in years since qualifying.

d. *Supplementary figure 4*: Part 2 mark in relation to Part 1 mark.

e. *Supplementary figure 5*: PACES mark in relation to Part 2 mark.

f. *Supplementary figure 6*: PACES mark in relation to Part 1 mark.

g. *Supplementary figure 7*: The fitted multivariate, multilevel model.

h. *Supplementary figure 8*: Correlations/covariances at the candidate and medical school levels.

i. *Supplementary figure 9*: Relationship between effects at the medical school level at Part 1, Part 2 and PACES.

Supplementary figure 1: Part 1 mark in relation to time in years since qualifying

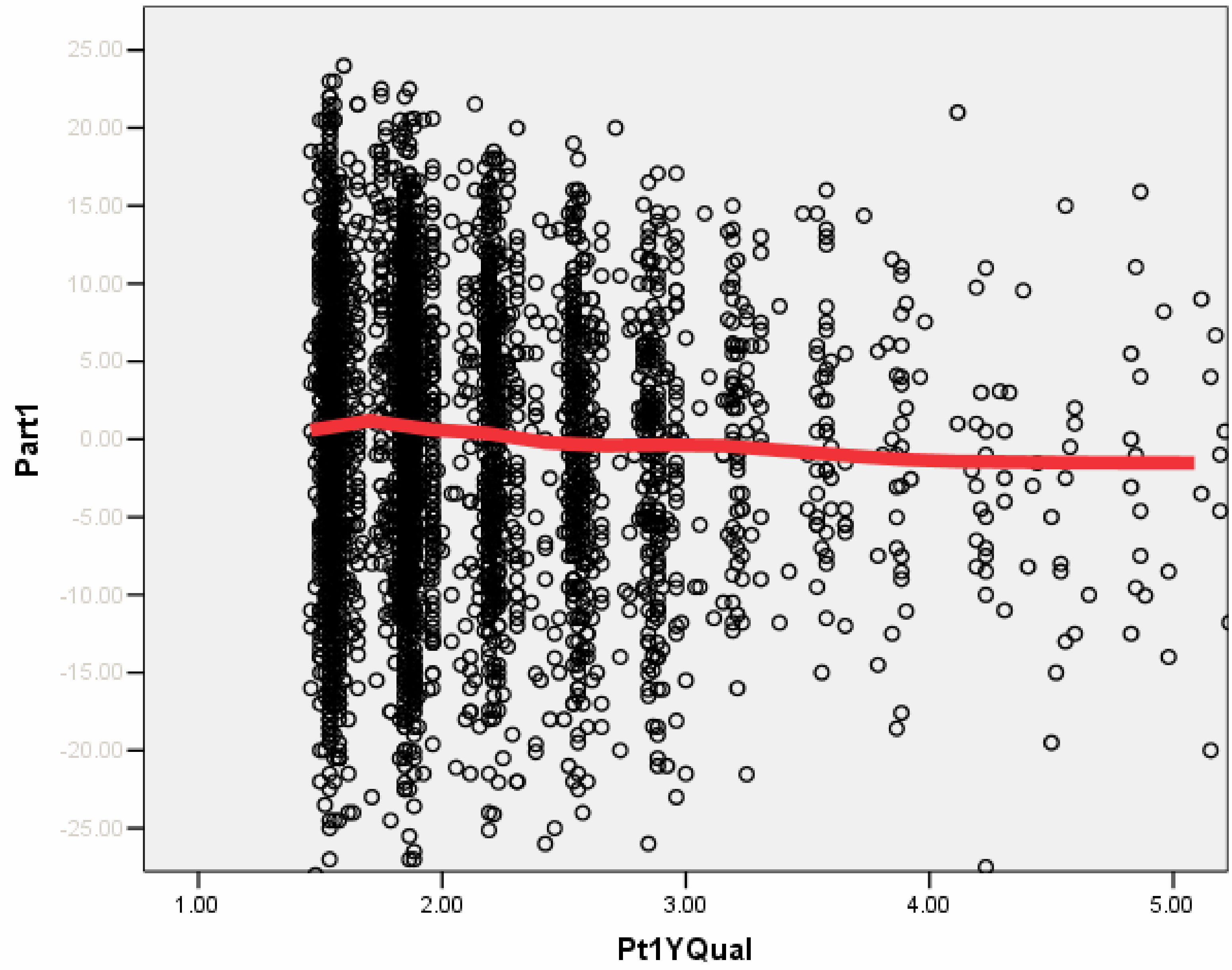

Note: A few points outside of the range of the axes have been omitted for clarity

Supplementary figure 2: Part 2 mark in relation to time in years since qualifying

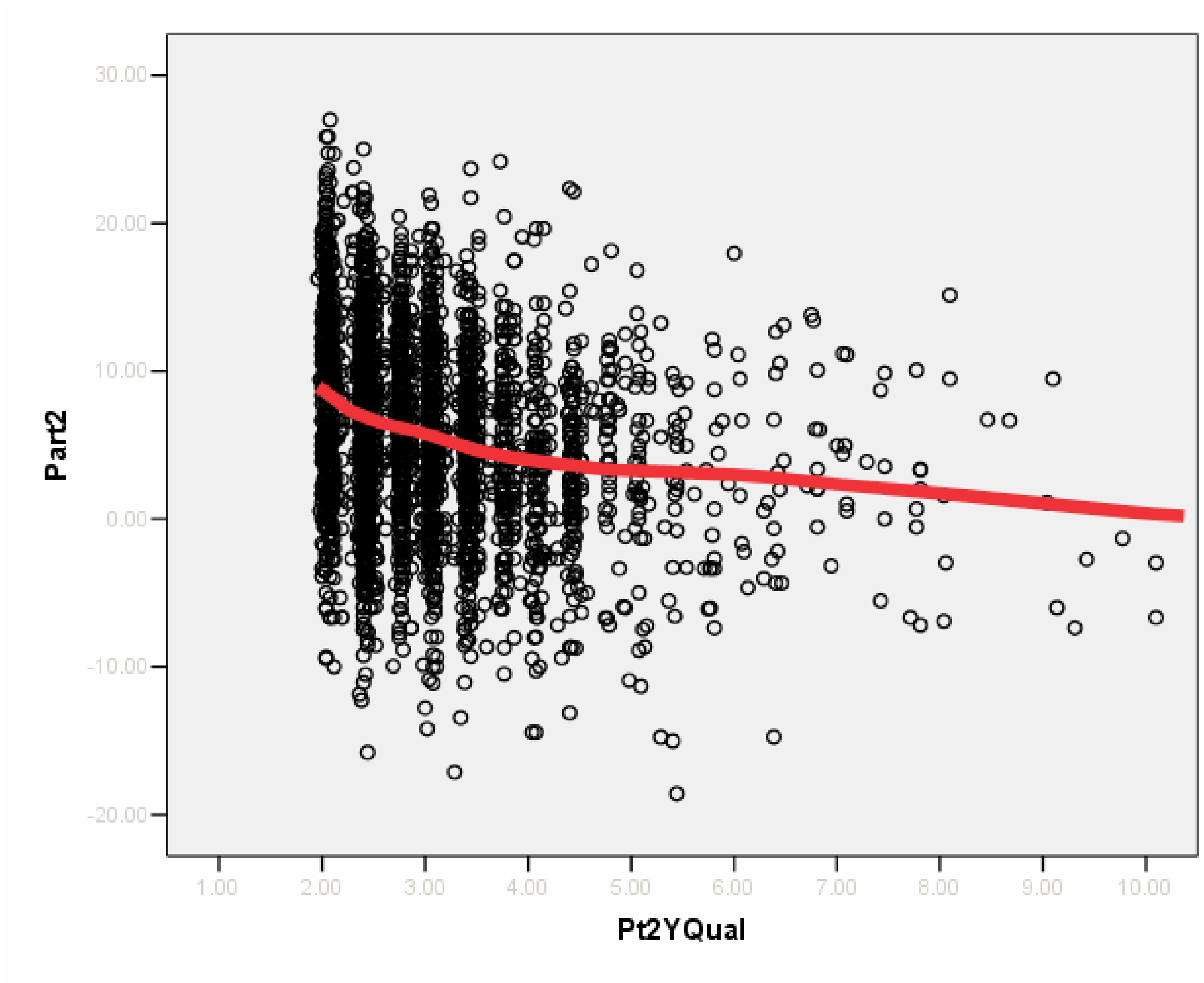

Note: A few points outside of the range of the axes have been omitted for clarity

Supplementary figure 3: PACES mark in relation to time in years since qualifying

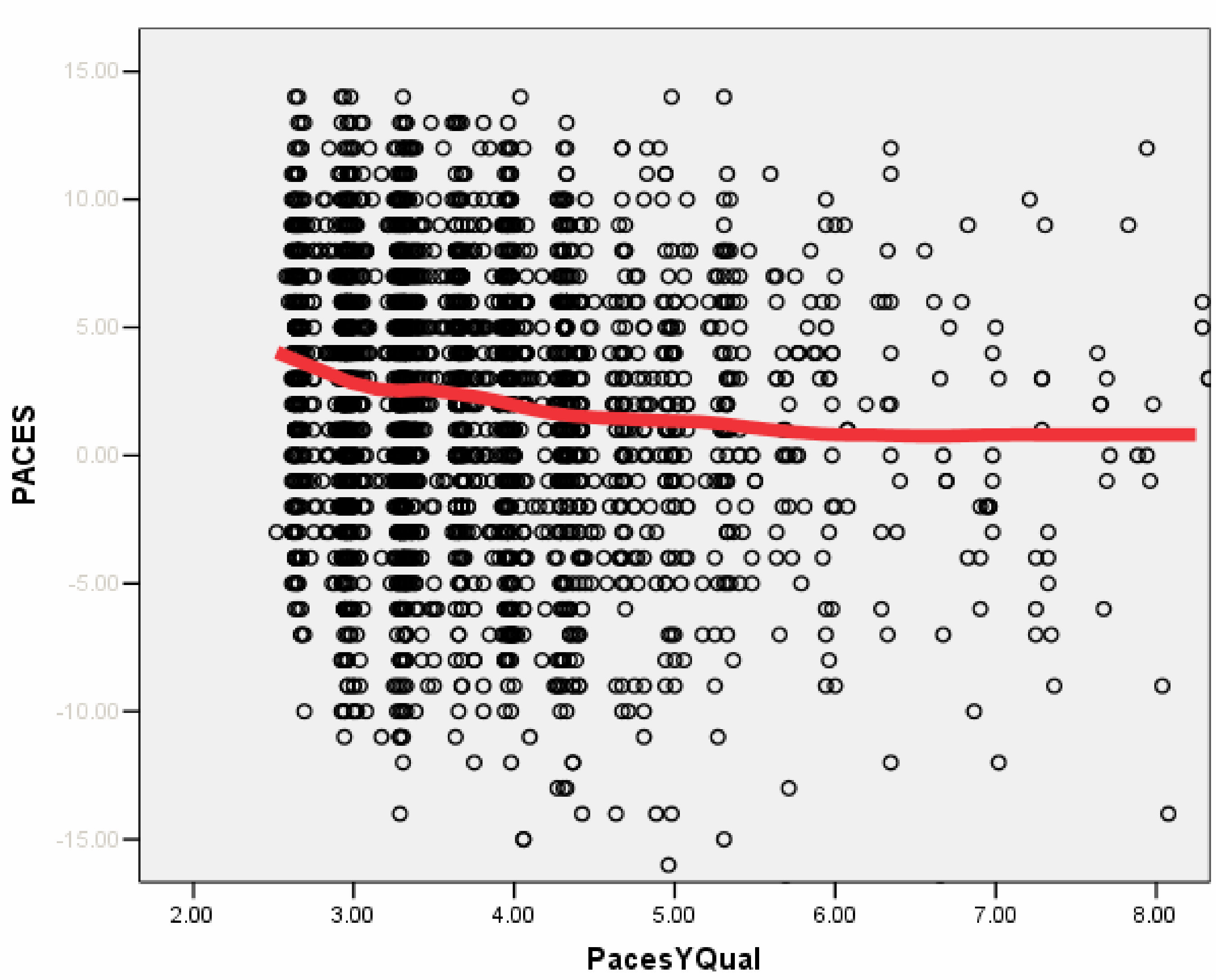

Note: A few points outside of the range of the axes have been omitted for clarity

Supplementary figure 4: Part 2 mark in relation to Part 1mark

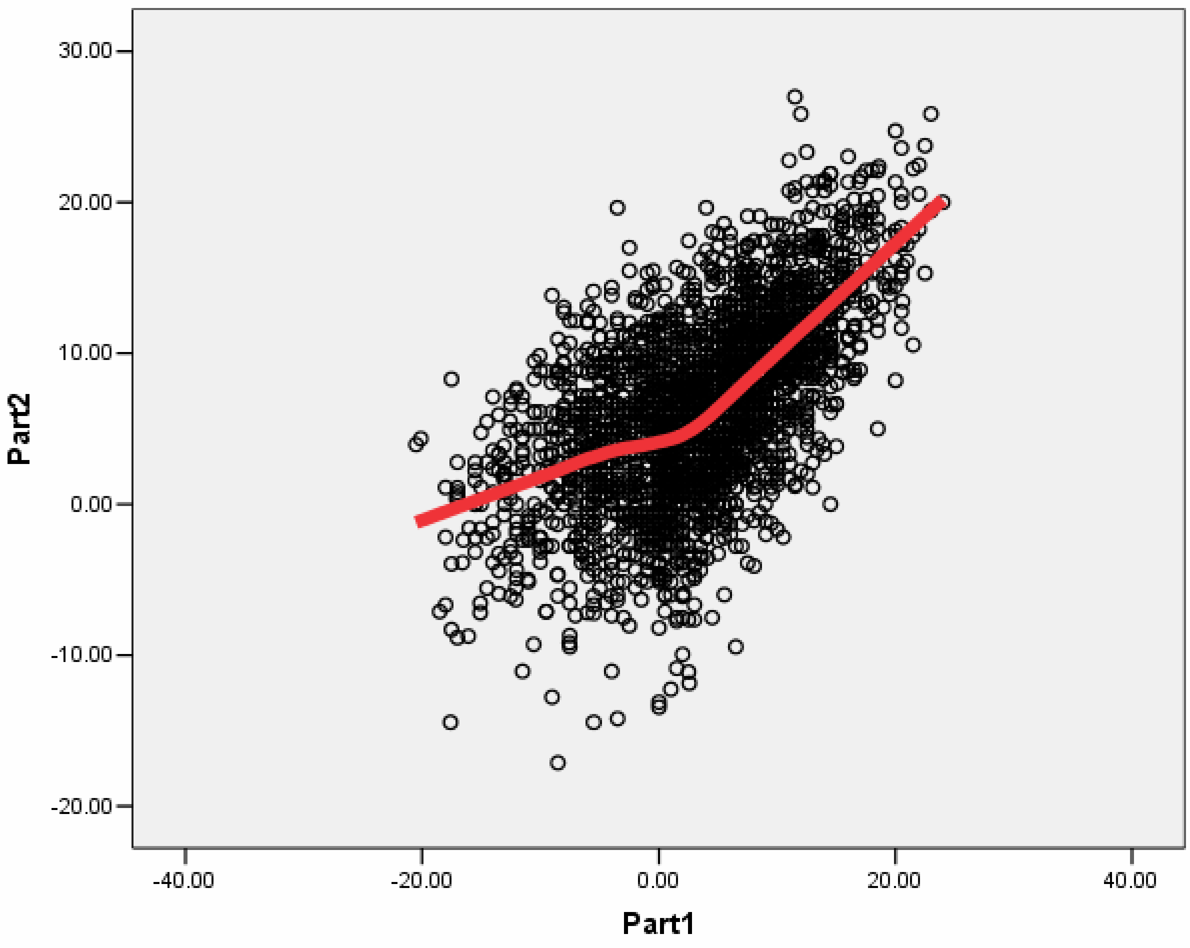

Supplementary figure 5: PACES mark in relation to Part 2mark

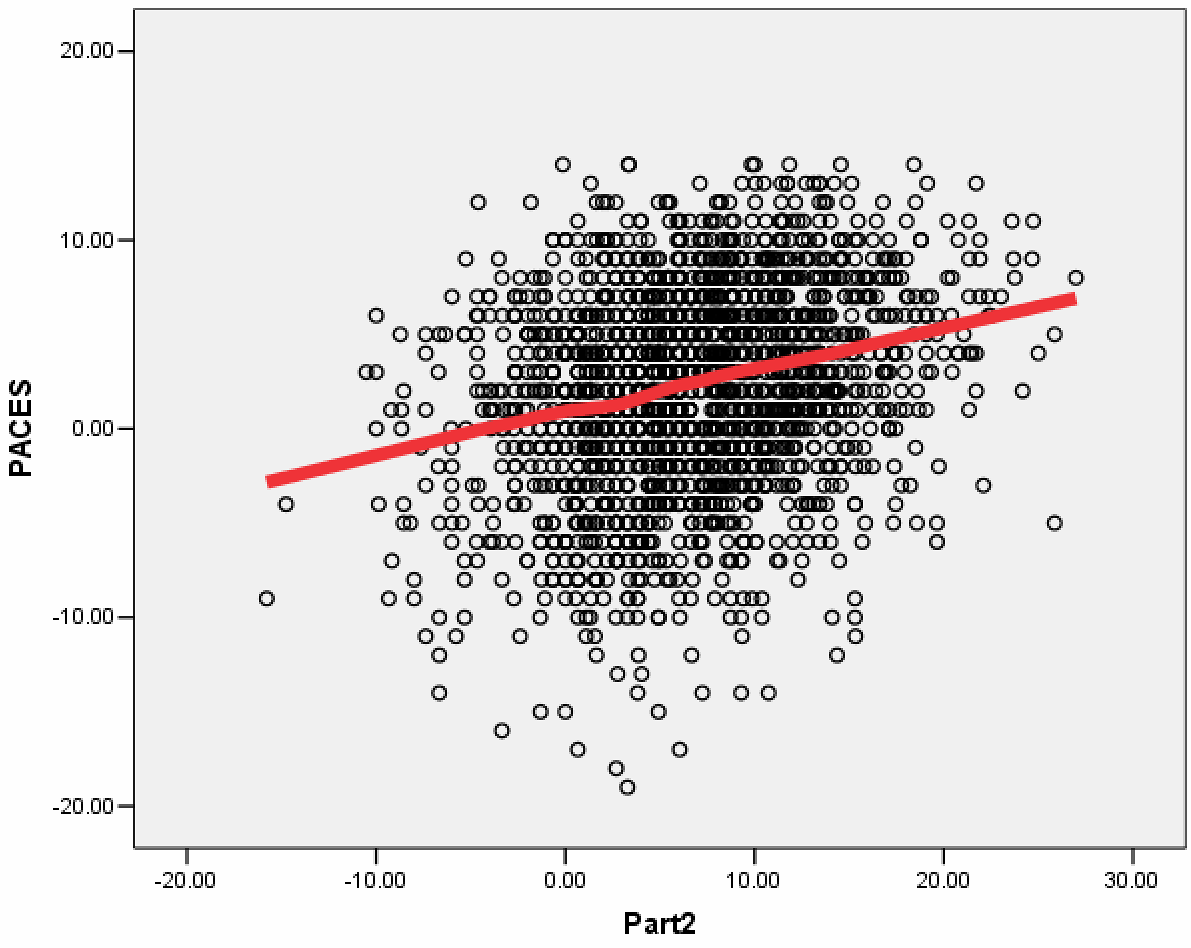

Supplementary figure 6: PACES mark in relation to Part 1mark

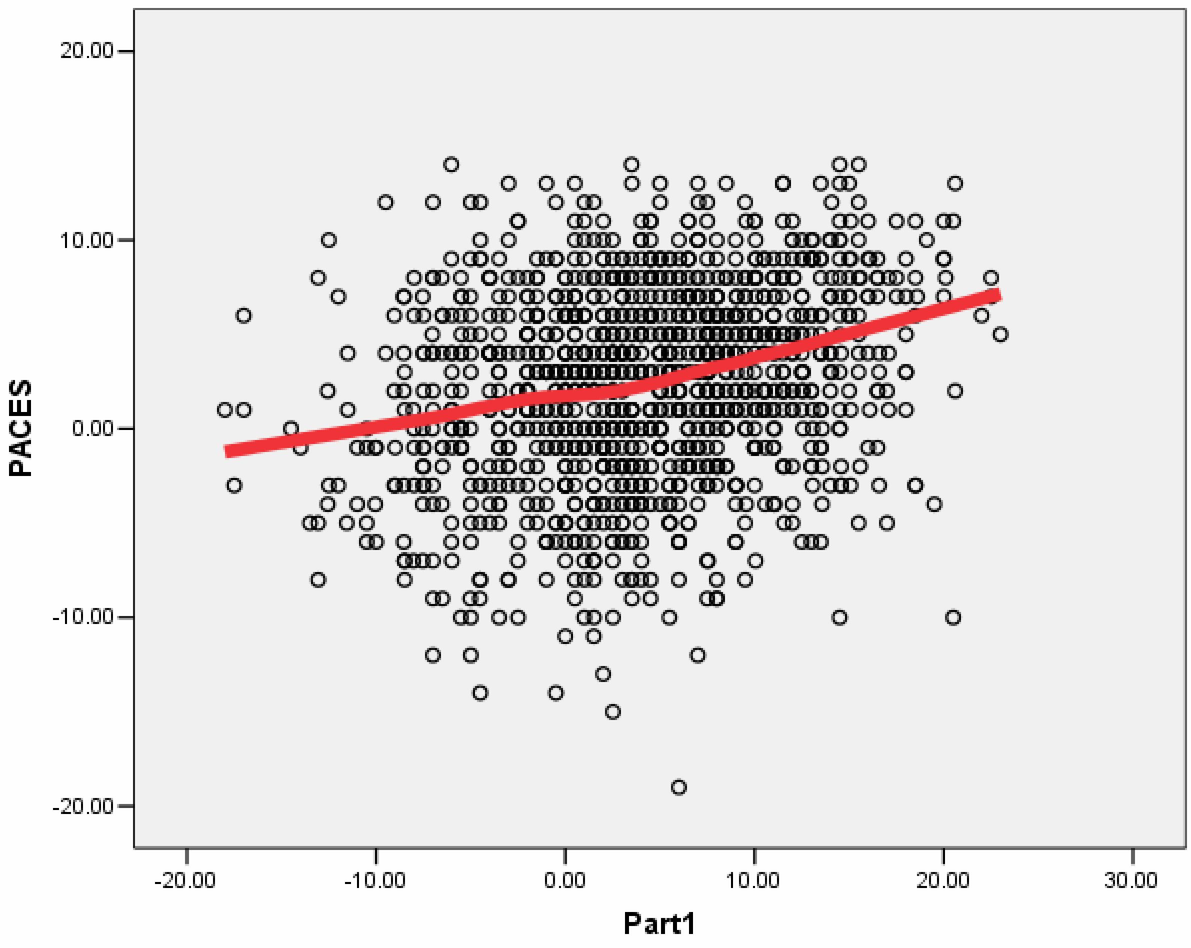

Supplementary figure 7: The fitted multivariate, multilevel model.

$$\text{resp}_{1jk} \sim \text{N}(XB, \Omega)$$
$$\text{resp}_{2jk} \sim \text{N}(XB, \Omega)$$
$$\text{resp}_{3jk} \sim \text{N}(XB, \Omega)$$

$$\text{resp}_{1jk} = \beta_{0jk}\text{cons.part1}_{ijk} + -0.756(0.264)\text{sex.part1}_{ijk} + 0.944(0.339)\text{white\_1.part1}_{ijk} + -1.035(0.303)\text{white\_9.part1}_{ijk} + -0.558(0.127)\text{Pt1yrqual.part1}_{ijk}$$

$$\beta_{0jk} = 2.726(0.866) + v_{0k} + u_{0jk}$$

$$\text{resp}_{2jk} = \beta_{1jk}\text{cons.part2}_{ijk} + -0.690(0.210)\text{sex.part2}_{ijk} + 0.681(0.266)\text{white\_1.part2}_{ijk} + -0.262(0.245)\text{white\_9.part2}_{ijk} + 0.043(0.005)\text{Pt2delay.part2}_{ijk} + -0.579(0.085)\text{Pt2yrqual.part2}_{ijk}$$

$$\beta_{1jk} = 5.843(0.595) + v_{1k} + u_{1jk}$$

$$\text{resp}_{3jk} = \beta_{2jk}\text{cons.paces}_{ijk} + 1.115(0.193)\text{sex.paces}_{ijk} + 1.027(0.237)\text{white\_1.paces}_{ijk} + -0.229(0.230)\text{white\_9.paces}_{ijk} + 0.011(0.010)\text{PACESdelay.paces}_{ijk} + -0.389(0.087)\text{PACESyrqual.paces}_{ijk}$$

$$\beta_{2jk} = 1.025(0.581) + v_{2k} + u_{2jk}$$

$$\begin{bmatrix} v_{0k} \\ v_{1k} \\ v_{2k} \end{bmatrix} \sim \text{N}(0, \Omega_v) : \Omega_v = \begin{bmatrix} 8.345(2.850) & & \\ 4.533(1.576) & 2.557(0.916) & \\ 2.148(0.849) & 1.272(0.491) & 0.787(0.327) \end{bmatrix}$$

$$\begin{bmatrix} u_{0jk} \\ u_{1jk} \\ u_{2jk} \end{bmatrix} \sim \text{N}(0, \Omega_u) : \Omega_u = \begin{bmatrix} 75.402(1.648) & & \\ 39.129(1.113) & 43.916(1.025) & \\ 12.027(1.121) & 9.565(0.747) & 27.234(0.718) \end{bmatrix}$$

Supplementary figure 8: Correlations/covariances at the candidate and medical school levels.

	cons.part1	cons.part2	cons.paces
cons.part1	75.402<br>(1.648)<br>Corr: 1.000		
cons.part2	39.129<br>(1.113)<br>Corr: 0.680	43.916<br>(1.025)<br>Corr: 1.000	
cons.paces	12.027<br>(1.121)<br>Corr: 0.265	9.565<br>(0.747)<br>Corr: 0.277	27.234<br>(0.718)<br>Corr: 1.000

	cons.part1	cons.part2	cons.paces
cons.part1	8.345<br>(2.850)<br>Corr: 1.000		
cons.part2	4.533<br>(1.576)<br>Corr: 0.981	2.557<br>(0.916)<br>Corr: 1.000	
cons.paces	2.148<br>(0.849)<br>Corr: 0.838	1.272<br>(0.491)<br>Corr: 0.897	0.787<br>(0.327)<br>Corr: 1.000

Supplementary figure 9: Relationship between effects at the medical school level at Part 1, Part 2 and PACES.

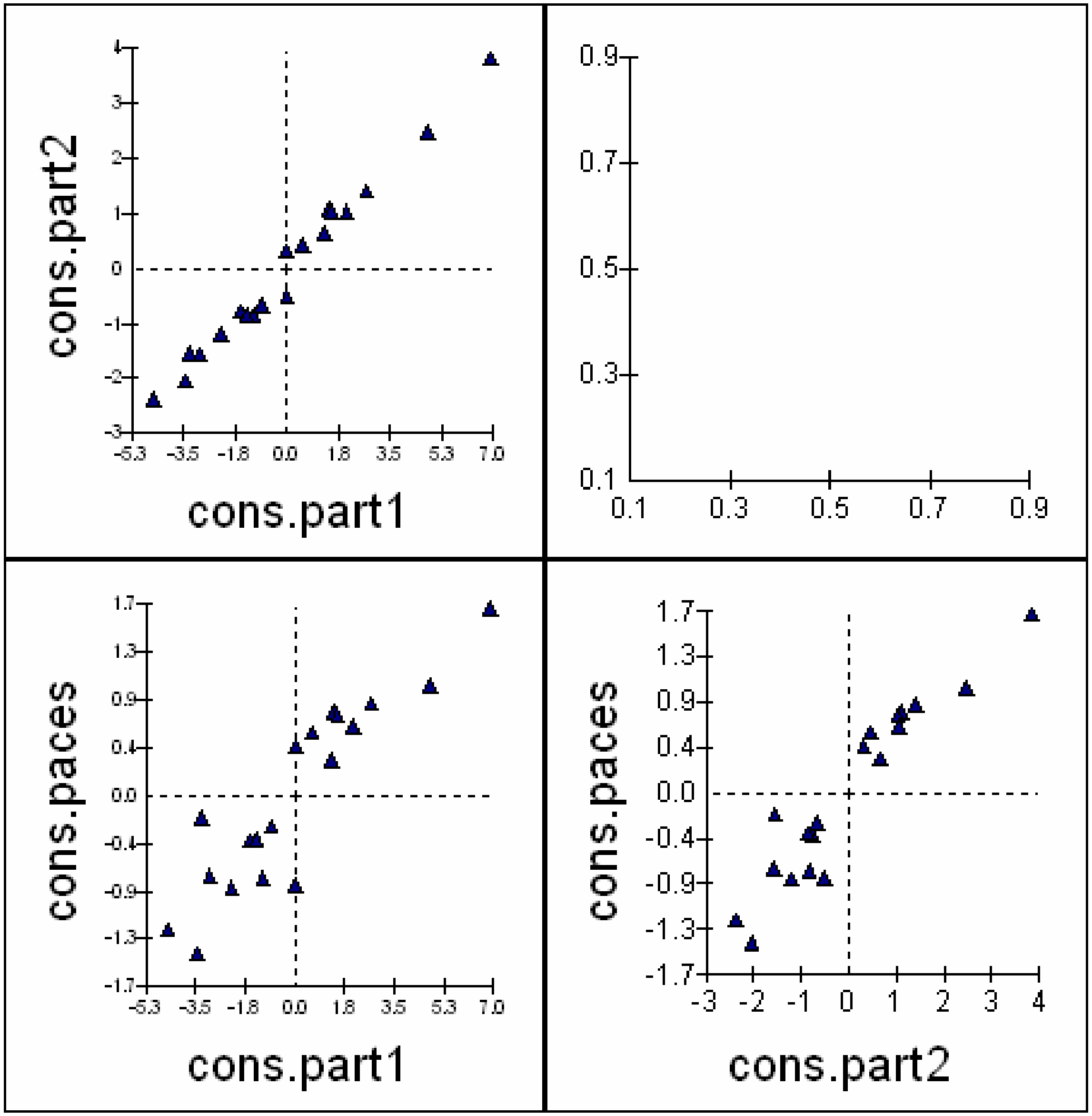

Supplementary figure 10: Mean score of all UK graduates taking MRCP(UK) between 1989 and 2005. Score indicates marks relative to the pass mark for the particular exam (shown as the horizontal line). Points are shown ± 1 SE.

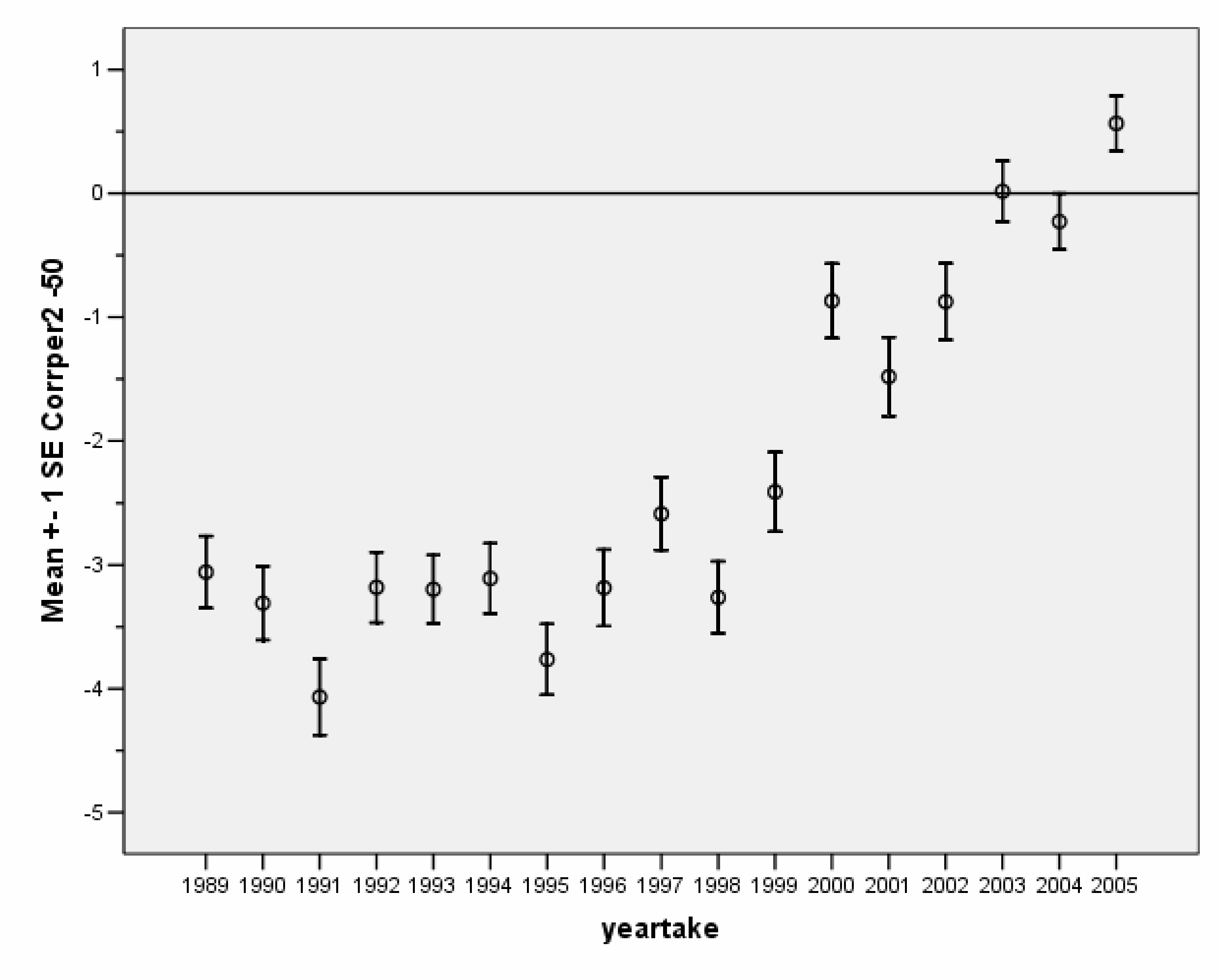

Supplementary figures 11a-11e: Mean MRCP(UK) score of graduates of individual medical schools 1989 and 2005. Note that scores have been standardised for overall changes in performance (see Supplementary Figure 10), and hence show differences from the overall average for all UK graduates (shown as the horizontal line at zer0). Points are shown ± 1 SE.

a) Aberdeen, Belfast, Birmingham and Bristol
b) Cambridge, Dundee, Edinburgh, and Glasgow
c) Leeds, Leicester, London and Liverpool
d) Manchester, Newcastle, Nottingham and Oxford
e) Sheffield, Southampton and Wales

***Supplementary figure 11a*:**

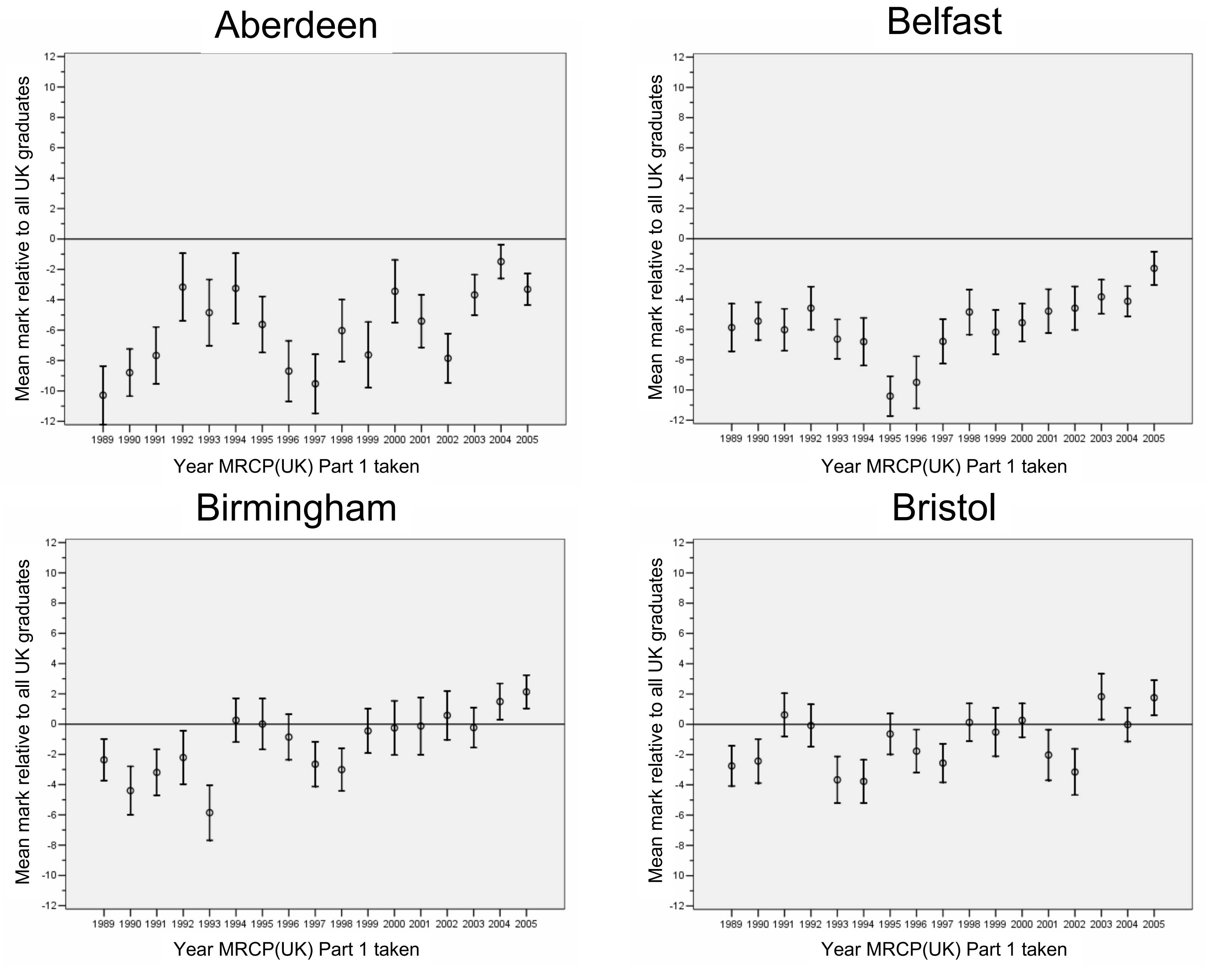

***Supplementary figure 11b*:**

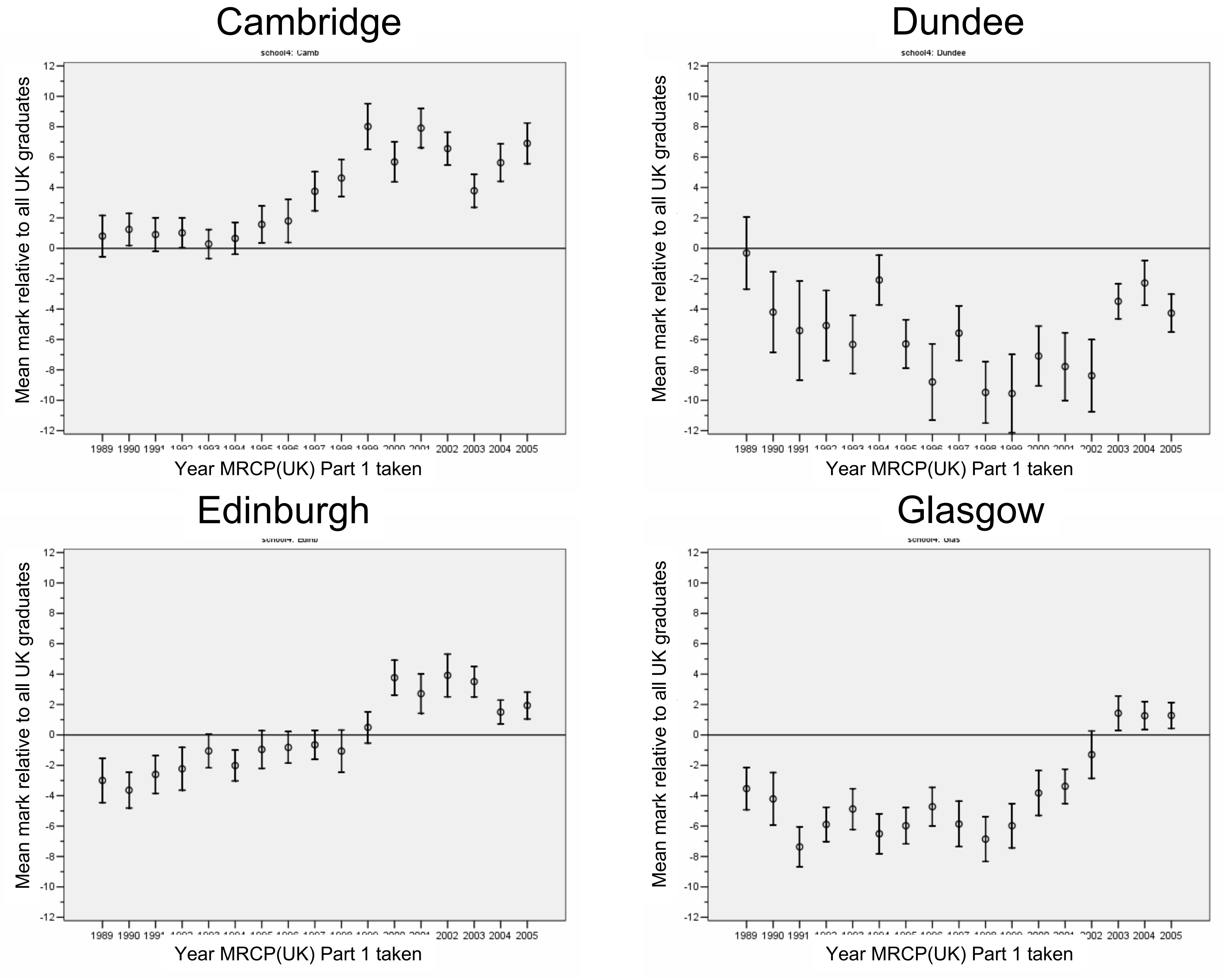

Supplementary figure 11c:

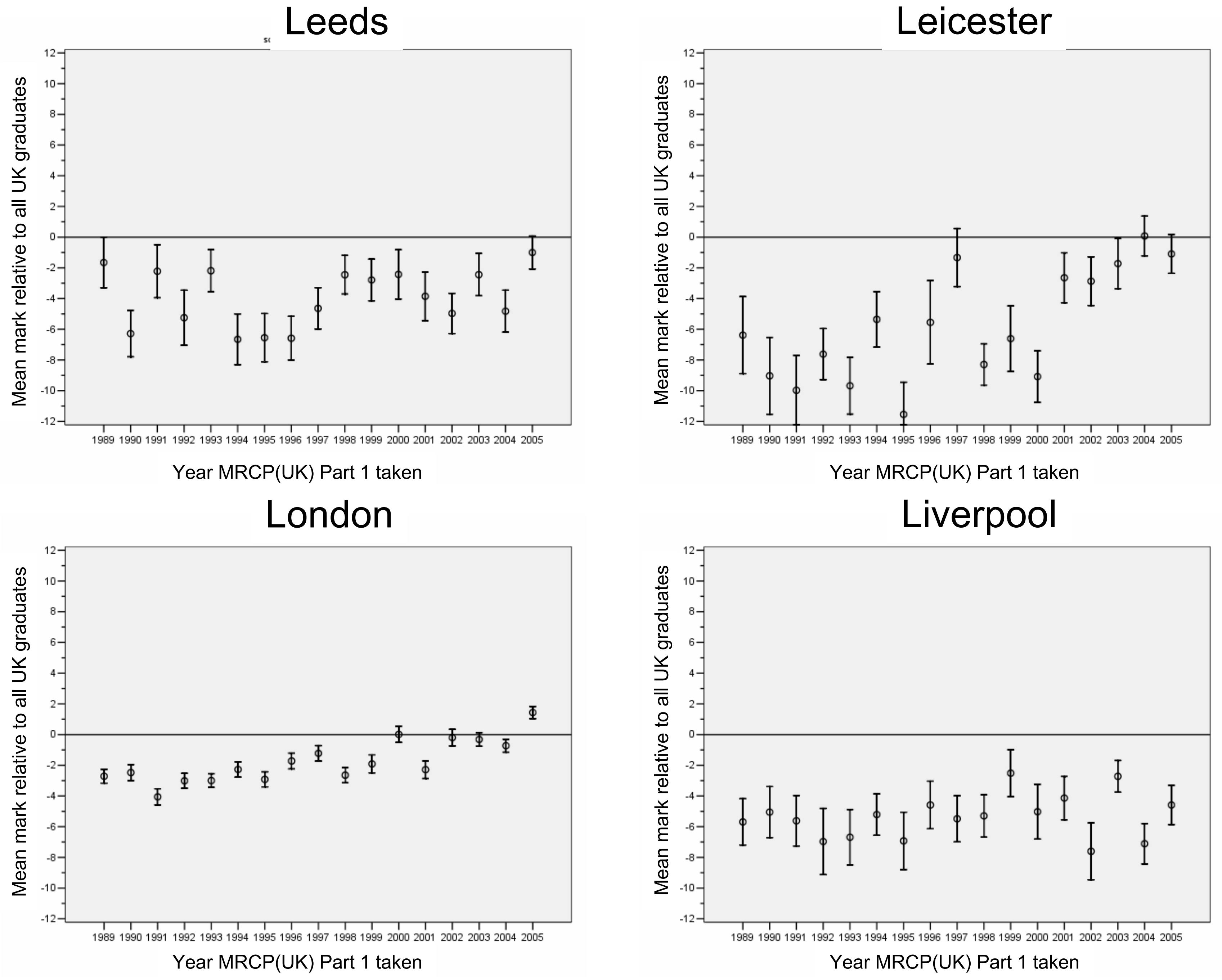

***Supplementary figure 11d*:**

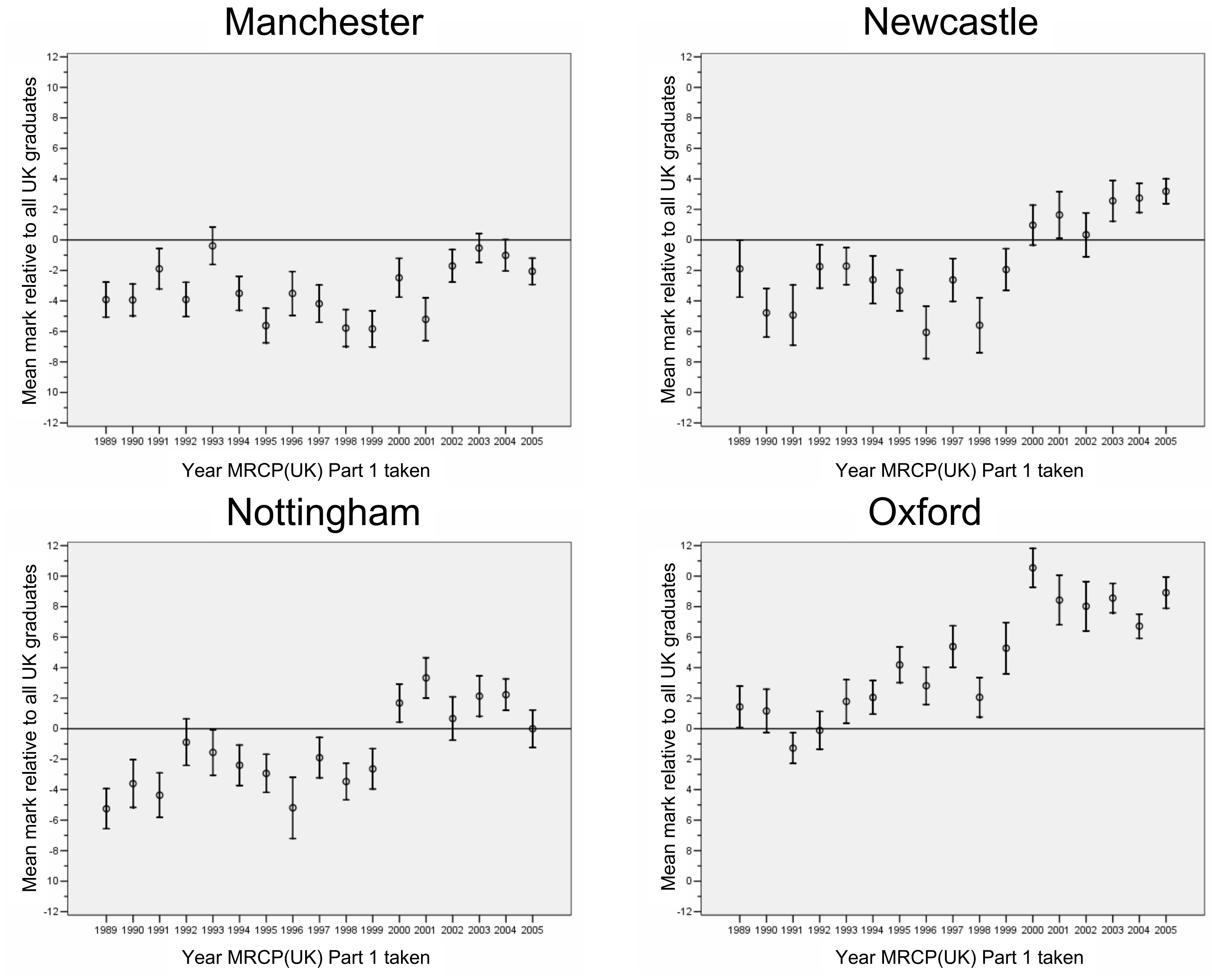

***Supplementary figure 11e*:**

Sheffield

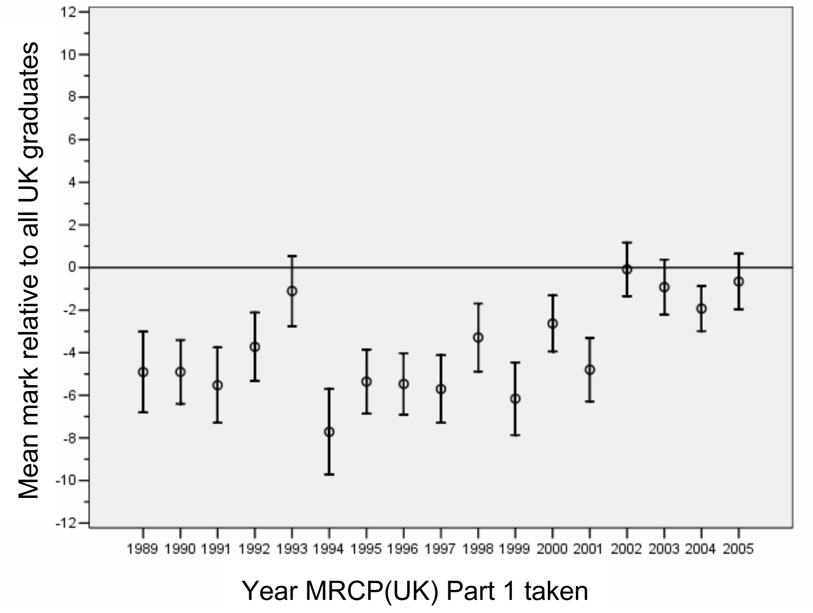

Southampton

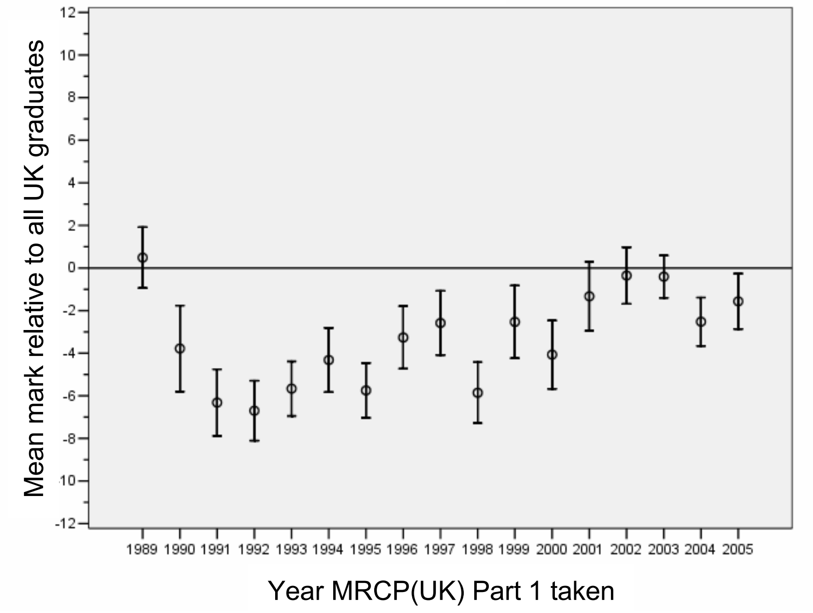

Wales

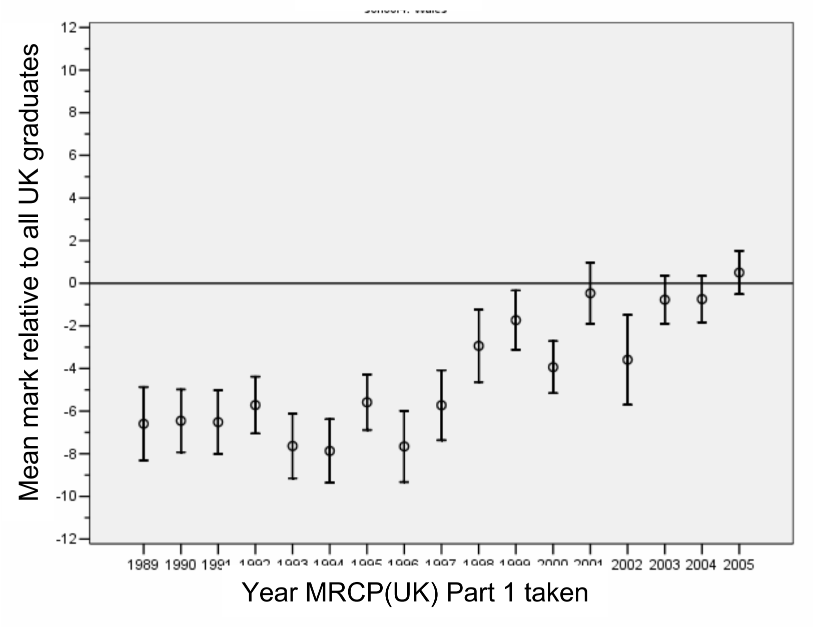

Supplement: Additional file 1 — Supplementary Information. Additional statistical analyses and graphs for individual medical schools. [file 1741-7015-6-5-S1.pdf]
